# Deep‐Learning‐Based Denoising for Improved Phase Precision in Electron Holography of Electromagnetic Fields in Nanoscale Materials

**DOI:** 10.1002/advs.77380

**Published:** 2026-08-31

**Authors:** Ye Luo, Luyang Wang, Zhiling Yu, Xiaowen Chen, Xiaoyu Wang, Ya‐Hui Jia, Thibaud Denneulin, Yu Han, Rafal E. Dunin‐Borkowski, Fengshan Zheng, Qi Liu

**Affiliations:** ^1^ School of Future Technology South China University of Technology Guangzhou China; ^2^ Center for Electron Microscopy South China University of Technology Guangzhou China; ^3^ Spin‐X Institute School of Physics and Optoelectronics Guangdong‐Hong Kong‐Macao Joint Laboratory of Optoelectronic and Magnetic Functional Materials South China University of Technology Guangzhou China; ^4^ School of Emergent Soft Matter South China University of Technology Guangzhou China; ^5^ Ernst Ruska‐Centre for Microscopy and Spectroscopy with Electrons Forschungszentrum Jülich Jülich Germany

**Keywords:** deep neural network, denoising, low dose conditions, weak phase signals

## Abstract

Electron holography is a powerful tool for the quantitative mapping of electric and magnetic fields at the nanoscale, with direct applications in semiconductor devices, magnetic nanostructures, and functional nanomaterials. However, its sensitivity is fundamentally limited by shot noise. At low dose conditions, shot noise overwhelms subtle fringe shifts that carry weak signals. Recent advances in deep learning have shown remarkable success in image denoising. Here we present HoloDenoiser, an unsupervised deep neural network that exploits the localization of phase information at sideband peaks in Fourier space as an explicit physics‐guided prior. The network achieves 3–4 fold improvement in phase precision across both Linear and Counting detector modes, outperforming conventional denoisers and state‐of‐the‐art deep learning baselines. We demonstrate the method experimentally on zero‐phase vacuum holograms and on built‐in potential mapping across a GaAs *p–n* junction. Using simulated holograms of a magnetic nano‐disk, we further recover weak magnetic phase signals with a ∼25‐fold reduction in the required electron dose. We anticipate that this work will provide a useful tool for the broader nanoscience community working on quantitative phase imaging at the nanoscale.

## Graphical abstract

Low‐dose electron holography is limited by shot noise, which buries weak phase signals. HoloDenoiser, a physics‐informed network that works simultaneously in the spatial and frequency domains, locates and protects the holographic sideband while suppressing noise in the hologram.

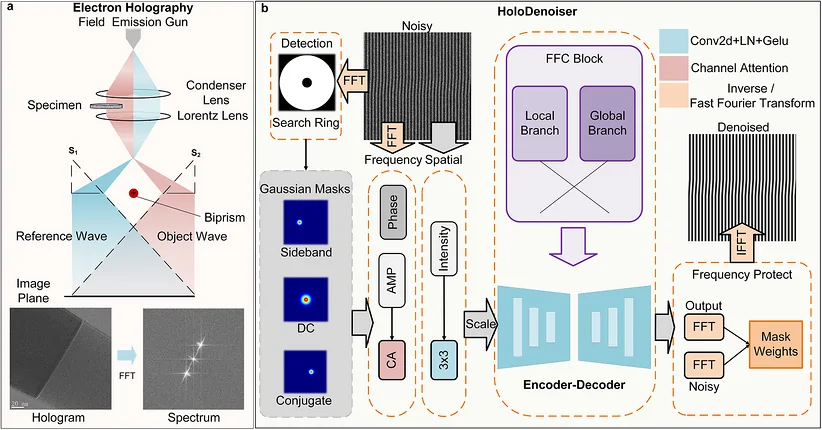

## 1 Introduction

Quantitative mapping of local electric and magnetic fields at the nanoscale is essential for understanding and engineering modern functional materials, including semiconductor devices, magnetic nanostructures, and energy materials. Among the available techniques, holography, conceived by Dennis Gabor in 1948, is uniquely capable of measuring the phase of the electron wave with nanometer spatial resolution, allowing direct retrieval of electrostatic and magnetic vector potentials [1, 2, 3]. By encoding phase into interference fringes through superposition with a reference wave, holography preserves the complete wavefront information (amplitude and phase) that is lost in conventional intensity imaging. The development of coherent sources—lasers for optical holography, field emission guns for electron holography, and synchrotrons for X‐ray holography—extended holographic imaging across vastly different wavelength regimes [4, 5, 6]. Building on this capability, electron holography has facilitated visualization of magnetic structures in materials, confirmation of the Aharonov–Bohm effect, observation of quantized vortex dynamics, and nanometer resolution mapping of potentials of semiconductors [7, 8, 9, 10, 11, 12, 13]. However, realizing the full quantitative potential of these measurements—particularly for weak phase signals such as those arising from single magnetic moments or buried interface charges—remains constrained by a fundamental noise limit that motivates the present work.

**FIGURE 1:**
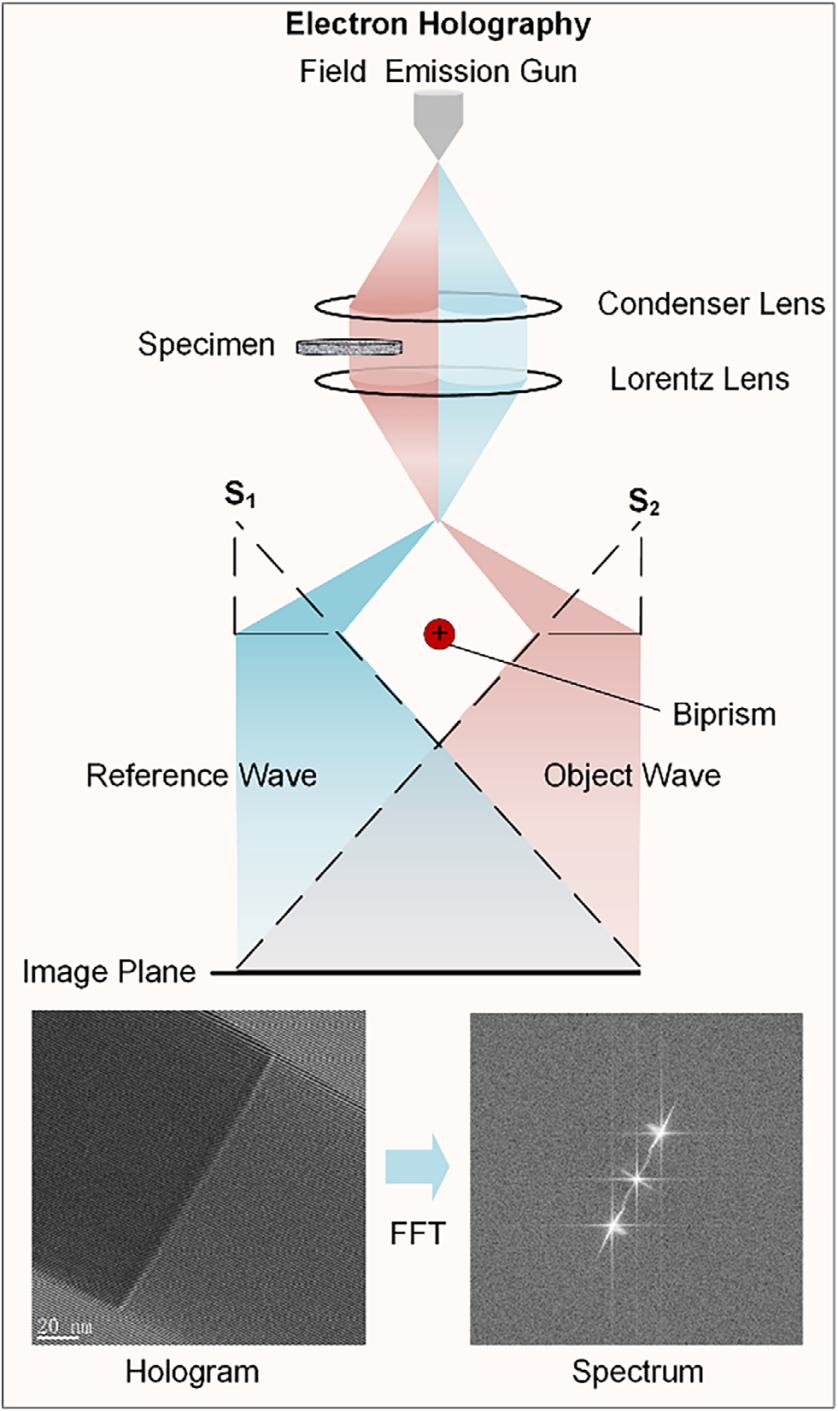
Representative holography with coherent electron sources in the transmission electron microscope. Coherent electrons from a field emission gun illuminate both the specimen and vacuum nearby. An electrostatic biprism attracts electrons to create overlap between the object wave transmitted through the specimen and a reference wave passing through the vacuum. Their interference at the image plane produces the hologram encoding the phase and amplitude of the object wave, which carries the electric and magnetic information of the specimen. The Fast‐Fourier‐transformed (FFT) of the hologram yields the spectrum, in which the center band (autocorrelation term) and two symmetric sidebands (+1 and −1 orders) are clearly separated, enabling reconstruction of the object wave's phase and amplitude.

In electron holography (Figure 1), a coherent electron beam from a field emission gun illuminates both the specimen and an adjacent vacuum region. An electrostatic biprism is used to attract electrons from both sides to create overlap between the object wave transmitted through the specimen and a reference wave passing through vacuum, producing interference patterns in real space. The resulting hologram contains periodic fringes whose local phase shifts encode the specimen's electrostatic and magnetic vector potentials. This imaging geometry produces a characteristic three‐band frequency structure (two sidebands and a center band) in reciprocal space, where phase information concentrates in sharply localized sidebands at the carrier frequency, while shot noise distributes uniformly across the entire Fourier spectrum. The phase sensitivity of electron holography is fundamentally limited by noise of distinctly different character than in conventional imaging. Shot noise arising from the discrete nature of electrons distributes uniformly across the Fourier spectrum and superimposes on the phase‐encoding sidebands, while readout noise, thermal fluctuations, and mechanical instabilities further degrade the recovered phase [14, 15]. Critically, these noise sources do not merely blur the hologram—they displace the interference fringes and thereby corrupt the encoded phase directly. This problem becomes particularly severe under the low dose conditions essential for beam‐sensitive specimens and for the detection of weak phase signals [16, 17]. For instance, detecting subtle signatures such as a single Bohr magneton requires phase precision of ∼10^−^^5^ radians—far exceeding standard capabilities [18]. Although long exposure times can suppress stochastic noise, it is susceptible to specimen and instrument drift and causes unavoidable radiation damage from high‐energy electrons [19, 20].

On the other hand, filtering in both the reciprocal and real spaces has also been applied effectively to hologram denoising [21, 22, 23]. In particular, frequency‐domain methods in the reciprocal space face a fundamental trade‐off: a narrow bandwidth degrades spatial resolution but suppresses high‐frequency noise. This highlights the fact that conventional filters in the Fourier space cannot effectively distinguish between the noise and signals in the overlapping regions, while filters in the real space may destroy the three‐band structure of interference fringes, thus leading to sideband distortions. The wavelet hidden Markov model (WHMM) [24, 25] partially addresses these limitations through scale‐aware statistical priors but relies on hand‐crafted parameters that limit generalization. Meanwhile, deep learning has revolutionized image restoration across microscopy [26, 27, 28, 29], with synthetic training data enabling broad generalization, and unsupervised approaches have obviated the requirement for the ground truth [30, 31, 32]. More recent deep learning denoisers—trained on natural or generic microscopy images—learn priors that may even be counterproductive for holograms: standard downsampling operations violate the Nyquist–Shannon criterion at the carrier frequency, folding high‐frequency content back onto the sidebands as aliasing artifacts [33, 34].

Here, we introduce HoloDenoiser, an unsupervised deep neural network dedicated to denoise electron holograms by exploiting their characteristic spectral structures, using off‐axis electron holography as the demonstration benchmark. We replace standard downsampling with *anti‐aliasing* operators throughout the networks, preventing the spectral folding that would otherwise contaminate the sharp sideband peaks critical for phase retrieval. Our method generalizes well across different camera modes, maintaining robust performance under both Counting and Linear detection regimes. In both cases, it reduces phase noise by a factor of 3–4 while preserving the inverse‐square‐root law, outperforming both conventional denoising and state‐of‐the‐art deep learning methods. For weak phase objects (∼0.05 radian), characteristic phase patterns can be recovered at an electron dose as low as just 15.43 e^−^/nm^2^ (pixel size = 0.36 nm), ∼25‐fold reduction in the required electron dose. Power spectral density analyses show that the network selectively suppresses broadband noise but preserves carrier‐frequency peaks. By preserving the underlying signal rather than merely attenuating amplitude, the network opens new possibilities for the quantitative characterization of beam‐sensitive functional nanomaterials.

The remainder of this paper is organized into five sections. We begin with Section 2, which offers a comprehensive review of existing denoising approaches, covering both traditional techniques and deep learning methods. Building on that, Section 3 presents our proposed methodology in detail. Section 4 then describes the holographic data used for training and provides the relevant training details. In Section 5, we report the denoising results obtained on both simulated and experimentally captured holograms, and we further include a power spectral density analysis to support the findings. Finally, Section 6 draws conclusions, discusses the limitations of the current method, and offers prospects for its further deployment and extension.

## 2 Related Work

### 2.1 Traditional Denoising Methods

Traditional denoising methods for electron holograms have largely relied on classical filtering methods (e.g., Wiener, median, and bilateral filters), patch‐based collaborative methods such as block‐matching and 3D filtering (BM3D), and variational regularization. Among these, the BM3D algorithm is widely recognized as a benchmark method for general‐purpose image denoising. In recent years, wavelet‐based methods such as the wavelet hidden Markov model (WHMM) have attracted particular attention in the field of electron holography. Midoh et al. [35] systematically compared the improvement in holographic phase estimation accuracy achieved by various conventional denoising methods under low electron dose conditions, demonstrating that WHMM achieves stable and superior performance. Tamaoka et al. [24] further applied WHMM denoising to the complex wave field during phase reconstruction of Nd_2_Fe_14_B samples, successfully suppressing phase discontinuities caused by dense fringes. Tomita et al. [36] extended WHMM to low dose electron holograms of weakly charged nanoparticles. Meanwhile, sparse coding and dictionary learning have also been applied to electron holograms [22]. Three‐dimensional tensor decomposition methods improve phase measurement accuracy by preserving the noise‐free principal components of stacked hologram series [37]. However, most of these methods rely on hand‐crafted priors or manually tuned parameters, limiting their adaptability across different datasets and noise levels.

### 2.2 Deep ‐ Learning‐Based Methods

In the field of deep learning‐based denoising, existing approaches can be categorized into supervised learning and self‐supervised/unsupervised learning according to training strategy. Supervised learning methods rely on paired noisy‐clean images for training and generally achieve high denoising performance. Mohan et al. [38] proposed a denoising framework called SBD, training convolutional neural networks (CNN) on simulated transmission electron microscopy (TEM) images and achieving favorable denoising results on both simulated platinum nanoparticles and experimental data. Lobato et al. [39] developed a CNN for restoring single‐shot electron microscopy images, outperforming BM3D by more than 6 dB in PSNR on scanning transmission electron microscopy (STEM) images. Ede et al. [40] introduced a dilated convolutional encoder‐decoder architecture to enhance the signal‐to‐noise ratio (SNR) of electron microscopy images. Ihara et al. [41] applied deep learning noise filtering to millisecond‐scale fast STEM imaging. However, in practical electron microscopy applications, noise‐free (ground‐truth) images are often difficult to obtain. Experimental electron microscopy images suffer from the combined interference of multiple factors, including readout noise, dark current, non‐uniform detector response, scan distortion, sample drift, and environmental vibration [39].

Self‐supervised and unsupervised methods do not require paired training data and learn denoising solely from noisy images themselves or from statistical relationships among noisy images [30, 42]. Noise2Void [32] employs a blind‐spot masking strategy to train on a single noisy image and has been successfully applied to STEM image denoising [43]. Noise2Atom [44] achieves effective denoising of STEM image sequences through an unsupervised architecture incorporating domain knowledge constraints. Kim et al. [45] proposed a self‐supervised framework that optimizes blind‐spot size by estimating the spatial correlation of noise, breaking the information limit across multiple TEM imaging modalities. Crozier et al. [31] applied unsupervised deep video denoising (UDVD) to in situ TEM videos of Pt/CeO_2_ nanoparticles, achieving a 40‐fold improvement in SNR and enabling the observation of surface structural dynamics at 10 ms temporal resolution.

The above methods have been predominantly developed and validated for conventional TEM and STEM imaging, where the recorded intensity typically serves as the final quantity of interest, and their applicability to electron holography remains largely unexplored.

## 3 Methodology

### 3.1 HoloDenoiser

**FIGURE 2:**
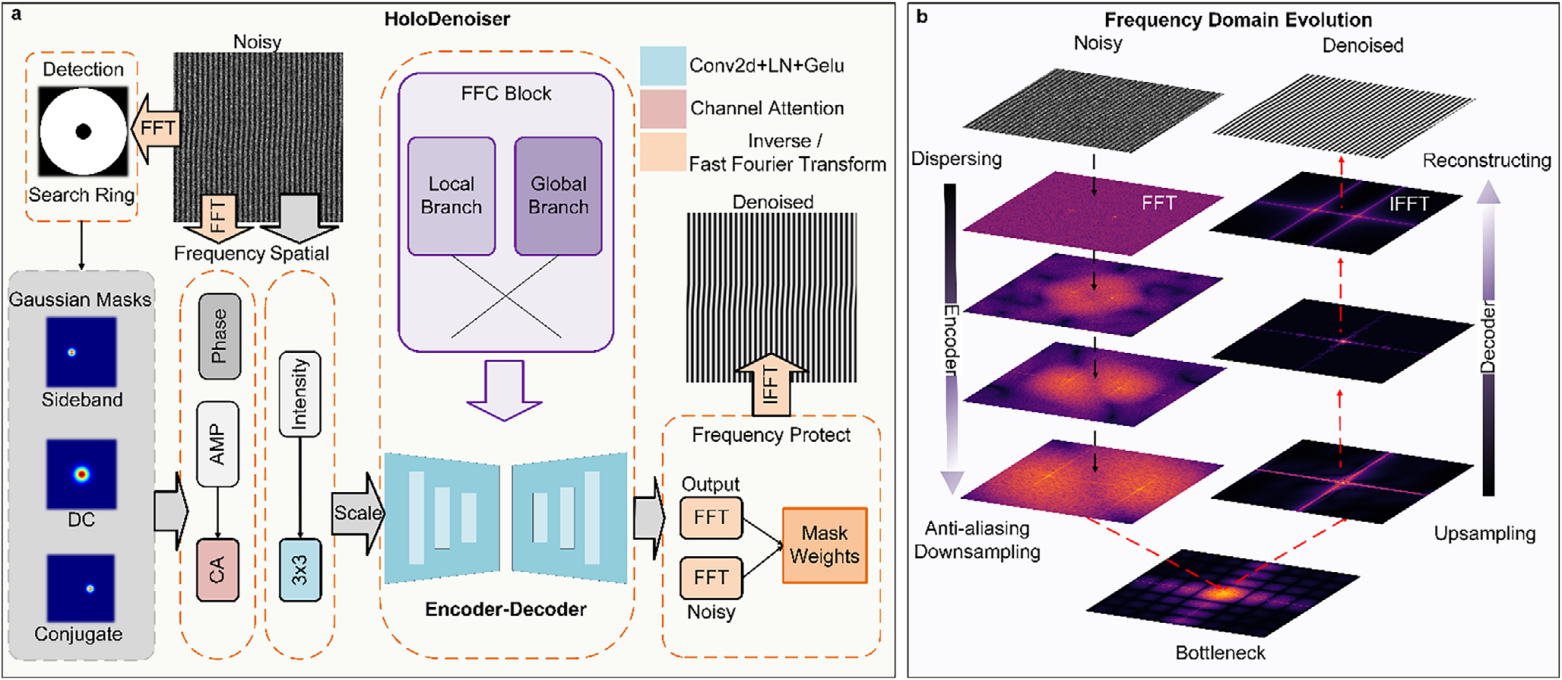
Deep learning for electron hologram denoising. (a) Architecture of HoloDenoiser. A noisy hologram is first transformed via FFT, from which a search ring automatically detects the sideband position. Based on this detection, Gaussian masks are generated for three frequency components: One sideband and its conjugate, the zero‐frequency (DC) component. The input is processed through dual pathways: (1) A frequency‐domain pathway that decomposes the FFT spectrum into the phase and amplitude (AMP), and (2) a spatial‐domain pathway that extracts intensity features. The combined mask serves as a spectral prior, guiding the frequency‐domain pathway through a channel attention (CA) mechanism that emphasizes information‐carrying frequency bands. These features are then combined through a scale mechanism and fed into an encoder–decoder network built upon Fast Fourier Convolution (FFC) blocks, each containing a local branch for spatial features and a global branch for frequency‐domain features. A frequency protect module is then applied to the pre‐computed Gaussian masks as weights in the Fourier domain to selectively preserve the sideband information while suppressing noise, yielding the final denoised hologram via inverse FFT (IFFT). (b), Frequency domain evolution through the encoder‐decoder network. The left branch shows the encoding path with *anti‐aliasing* downsampling, progressively extracting multiscale features. The right branch illustrates the decoding path that reconstructs the denoised hologram through upsampling operations. In the bottom branch, the bottleneck layer processes the most compressed representation while preserving essential frequency information.

HoloDenoiser is an end‐to‐end neural network framework for hologram denoising (Figure 2a). We first construct simulated holograms corrupted by Poisson noise to mimic the shot noise characteristics of modern electron detectors. Pairs of holograms are also generated by two independent Poisson samplings as a function of electron dose, spacing, and orientation of fringes to enable unsupervised training (see Section 4.1 Hologram Simulations for details).

The architecture is designed around the spectral characteristics of off‐axis holograms, where phase information concentrates in localized sidebands while noise distributes uniformly across the spectrum. As illustrated in Figure 2a, given an input noisy hologram, the network first identifies sideband and zero‐frequency (DC) locations in the reciprocal space via fast‐Fourier‐transform (FFT), and then places Gaussian masks that separate the encoded information around the sideband from the conventional signal around the center‐band. These masks serve as spectral priors that guide, but do not constrain, the learned features. Feature extraction proceeds through parallel frequency and spatial pathways. The frequency pathway first extracts global statistical descriptors from the spectral distributions of the signal and background regions. A channel attention (CA) module then turns these descriptors into channel‐wise modulation factors. The spatial pathway processes the original intensity image through convolutions, preserving fine‐scale fringe details. The scale scheme links the two streams, using Fourier‐domain statistics and the sideband mask to generate channel‐wise and spatial modulation terms. This modulation enters as a confidence‐weighted residual correction ahead of the encoder‐decoder backbone, letting the sideband priors guide feature extraction without overriding the original spatial representation.

These features are fed into an encoder–decoder backbone (see Section S1.3 HoloDenoiser architecture and Figure S1 for details) employing fast Fourier convolution (FFC) blocks [46]. The FFC blocks contain two interacting branches: a local branch performing spatial convolutions for fine‐grained texture processing, and a global branch operating through a Fourier Unit with learnable spectral modulation. This dual‐branch design captures the fringe patterns that conventional local convolutions require extensive layer stacking to represent. Overlapping window attention with Gaussian boundary weighting captures long‐range dependencies at the bottleneck, while the decoder employs skip‐connections enhanced by cross‐attention. The Frequency Protect module first blends complex‐valued spectra, then applies a small amplitude‐preservation correction in the detected sideband regions. Both the original noisy hologram and network output are transformed to the Fourier domain, where learnable mask weights balance contributions from each source: sideband regions retain higher contribution from the original signal to preserve phase integrity, while noise‐dominated regions adopt the denoised result.

### 3.2 Spectral Evolution and *Anti‐Aliasing*

To elucidate how the network achieves signal‐noise separation, we track spectral evolution across network stages (see Figure 2b). The 2D FFT spectrum of the input hologram exhibits the characteristic structure of holograms: Symmetric sideband peaks at the carrier frequency, a central DC peak encoding mean intensity, and a uniformly dispersed background representing the noise. As the encoder propagates, non‐structural frequency components gradually attenuate while the sideband peaks intensify. This demonstrates the network's capacity to learn discriminative features associated with primary fringe frequencies.

According to the Nyquist–Shannon sampling theorem, a signal can be perfectly reconstructed only if it is sampled at a rate at least twice its highest frequency component. When a feature map is downsampled by a factor of two, the Nyquist limit is halved accordingly, and any frequency content above this new limit cannot be represented at the reduced resolution. Instead of being discarded, these components fold back and reappear as spurious low‐frequency signals, a phenomenon known as *aliasing*. In conventional deep learning architectures, stride convolutions and pooling operations reduce spatial resolution without applying an appropriate low‐pass filter: stride convolutions apply a learned kernel and subsample the output in a single step, while pooling operations such as max pooling or average pooling aggregate local regions and retain only one value per window, neither of which explicitly attenuates frequencies above the new Nyquist limit prior to subsampling [33].

Conventional denoising introduces only minor spectral distortions, with slight texture loss but no significant quality degradation. Holograms, however, are fundamentally different. Phase reconstruction operates in reciprocal space, where a sideband is isolated via an aperture mask and inverse‐Fourier‐transformed to recover the complex wavefunction. The phase information is sharply localized at the carrier frequency, making any spectral leakage disproportionately damaging: when *aliasing* populates the vicinity of the sideband, a larger reconstruction aperture—desirable for higher spatial resolution—inevitably encompasses these spurious components, introducing artifacts into the reconstructed phase. HoloDenoiser addresses this by incorporating *anti‐aliasing* modules throughout the encoder–decoder pathway. Using the same network architecture, models with conventional downsampling exhibit pronounced high‐frequency leakage and sideband distortion, whereas the anti‐aliasing approach maintains a clean spectral distribution with well‐preserved carrier frequency peaks (see Section S2.1 Downsampling Strategy Comparison and Figure S3). The bottleneck stage then performs spectral purification, enhancing main frequency peak concentration while suppressing background noise. During the decoder stages, primary frequency components are robustly preserved without diffusion artifacts. Note that given the critical role of fringe continuity in phase reconstruction, we have also developed a Normalized Multi‐Component (NMC) loss that integrates spatial and frequency‐domain constraints to preserve high‐fidelity fringe structures (see Section S1.4 Loss).

### 3.3 Fast Fourier Convolution

Standard convolutions with small kernels are inefficient at capturing the long‐range periodicity of holographic fringes, whose spatial frequency is set by the biprism voltage. To jointly preserve fine fringe details and suppress spectrally uniform shot noise, we adopt Fast Fourier Convolution (FFC) [46] as the core building block of our encoder–decoder backbone. FFC splits the input feature map *X* ∈ *R* ^*H* × *W* × *C*^ along the channel axis into a local part *X^l^* and a global part *X^g^*, controlled by a ratio α ∈ [0, 1]. The local branch applies spatial convolution for fine‐grained textures; the global branch operates through a spectral transformer in the Fourier domain, achieving a full‐resolution receptive field. Cross‐branch transitions fuse information across scales:

(1) $$\begin{array}{c} \;Y^{l}=f_{l}\;\left(X^{l}\right)+f_{g\to l}\left(X^{g}\right) \end{array}$$

(2) $$\begin{array}{c} \;Y^{g}=f_{g}\;\left(X^{g}\right)+f_{l\to g}\left(X^{l}\right) \end{array}$$

(3) $$\begin{array}{c} z=F^{-1}\;\left(\varphi \left(F\left(x\right)\right)\right) \end{array}$$

where *f_l_* denotes a standard 3×3 convolution on the local channels, *f_g_* the spectral transformer on the global channel, and *f*_*g* → *l*_, *f*_*l* → *g*_ are 1×1 inter‐path convolutions that transfer information between the two paths. The core of *f_g_* is built around a Fourier Unit (3). Here $F/F^{-1}$ denote the 2‐D FFT and its inverse, and φ is a learnable spectral modulation (1×1 Conv–LN–ReLU). Because the sideband peaks that encode phase information are localized in reciprocal space, the Fourier unit can selectively preserve these features while attenuating broadband noise.

### 3.4 Phase Reconstruction

Phase images were reconstructed from the holograms by the standard Fourier‐space approach. The holographic sideband was pre‐filtered with an eighth‐order Butterworth filter, with an aperture size of about one third of the carrier frequency. For a fair comparison, the noisy object hologram was reconstructed together with its noisy reference hologram, and the denoised object hologram together with its denoised reference hologram, each reference taken from the corresponding vacuum region. Both paths therefore share the same sideband extraction and the same aperture size, any difference in the reconstructed phase comes from the denoising of the holograms themselves and not from a change in reconstruction bandwidth.

## 4 Dataset

### 4.1 Hologram Simulations

We simulated holograms using an ideal interference model and superimposed dose‐dependent Poisson noise to replicate the signal‐dependent noise characteristics of real imaging systems. This approach simultaneously generated noisy–noisy pairs for unsupervised training and noisy–clean pairs for quantitative evaluation.

Hologram intensity distributions were simulated using an ideal interference model Equation (4). Here, *x* and *y* denote coordinates perpendicular and parallel to the fringe direction in the image plane, respectively. *A_o_* (*x*, *y*) and *A_r_* (*x*, *y*) denote the object and reference wave amplitudes, respectively, and specimen‐induced phase modulation φ(*x*,  *y*), while the carrier frequency *q_c_* controls fringe spacing and θ determines fringe orientation. Fringe contrast is characterized by *V*. Electron dose (*N_e_*: dose rate × exposure time) was varied to control signal levels, and dose‐dependent Poisson noise was applied using Python's Poisson sampling function to replicate shot noise characteristics of real electron detection.

To simulate Linear (integrating) mode holograms, three degradation components were applied at the intensity level: Modulation transfer function (MTF) broadening via Gaussian convolution, analog‐to‐digital conversion with calibrated gain g ≈ 36.4 analog‐to‐digital unit (ADU)/electron, and additive Gaussian readout noise (σ ≈ 18 ADU, ∼0.5 electrons).

(4) $$\begin{array}{cc} I\;\left(x,y\right) & =N_{e}[A_{o}{\left(x,y\right)}^{2}+A_{r}{\left(x,y\right)}^{2}+2A_{o}A_{r}\left(x,y\right)V\\ & \;\times \;\cos\left(2\pi q_{c}\left(x\;\cos\;\theta +y\;\sin\;\theta \right)+\varphi \left(x,y\right)\right)] \end{array}$$

### 4.2 Experimental Hologram Acquisition

Vacuum off‐axis electron holograms for denoising validation were acquired using a direct electron Gatan K2‐IS camera operating in both Linear and Counting modes. A systematic test set of hologram pairs was collected by combining two dose rates (10 and 20 e^−^/pixel/s, pixel size = 0.36 nm) with ten exposure times (0.1, 0.2, 0.4, 0.8, 1.0, 2.0, 4.0, 6.0, 8.0, and 10.0 s), spanning a dose range of 1–200 e^−^/pixel, with an interference fringe spacing of 2.4 nm (∼6.9 pixels). Holograms of a GaAs *p–n* junction were acquired in Counting mode at a dose rate of 5 e^−^/pixel/s with an exposure time of 6 s (pixel size = 0.21 nm), yielding an interference fringe spacing of 1.33 nm (∼6.32 pixels). Reference holograms were recorded from a region of vacuum with the specimen removed from the field of view.

### 4.3 Training Strategy

We build on the Noise2Noise framework, which learns from two independent noisy observations of the same underlying signal and needs no clean reference. Such pairs are also cheap to obtain in practice, since two consecutive exposures of the same field of view already provide one.

Each clean hologram was corrupted by two independent noise realizations to form a single noisy–noisy pair, and the resulting pool was split into training, validation, and test sets at 80/10/10. The noise level is set directly by electron dose. Dose rates covered 10 to 40 e^−^/pixel/s, exposure times 0.1 to 10 s, yielding an accumulated dose of 1 to 400 e^−^/pixel under Poisson statistics. For Linear mode, we additionally applied the detector‐specific degradations detailed in the Hologram Simulations section. Fringe spacing and orientation were varied throughout, with image size fixed at 512 × 512 pixels. Because each noise realization is drawn independently, one clean hologram at a given dose can seed many distinct pairs, which we used to broaden the dataset.

For the simulated *p–n* junction experiment, approximately 5000 training images were generated. For experimental vacuum holograms, we adopted a two‐stage scheme. Each detector mode (Linear and Counting) was first pretrained on about 10 000 simulated pairs built with its own noise model, then fine‐tuned on about 2000 experimental holograms. For weak‐phase magnetic imaging, we pretrained on the vacuum data and fine‐tuned on about 3000 simulated magnetic structures. These comprised homogeneously magnetized disks, which produce an extended dipolar stray field, and vortex disks, which confine the signal to a localized core, with phase amplitudes spanning roughly 0.01 to 3 rad.

Some experimental holograms are large, such as 3838 × 3710 pixels, so they were tiled into 512 × 512 patches using a sliding window with 50% overlap. The translation equivariance of convolutions lets a model trained on fixed‐size patches run directly on full‐resolution images at inference. Data augmentation was limited to orthogonal rotations of 90°, 180°, and 270°, since interpolating continuous rotations would blur the fringes and compromise their integrity. All models were trained for 120 epochs with the Adam optimizer and a cosine‐annealed learning rate from 1 × 10^−^^3^ to 1 × 10^−^^6^. Early stopping was triggered after 5 epochs of no validation improvement. Training was performed on a single NVIDIA A100 GPU with 40 GB memory.

## 5 Results and Discussion

### 5.1 Evaluation on Simulated Datasets

**FIGURE 3:**
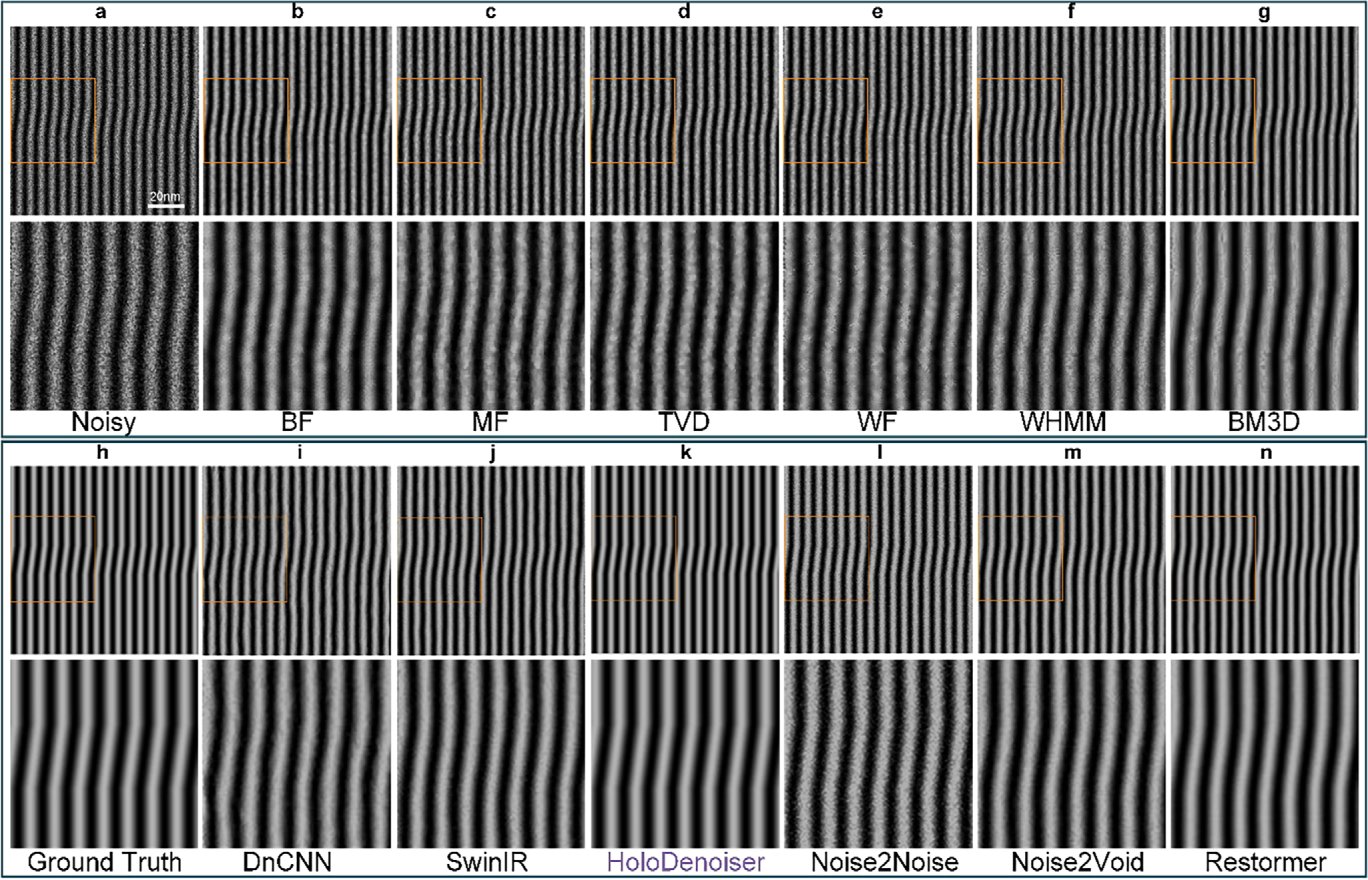
Denoising performance comparison of simulated *p–n* junction on holograms. Upper panel: noisy input (dose = 10 e^−^/pixel) and results from conventional methods including bilateral filter (BF), median filter (MF), total variation denoising (TVD), Wiener filter (WF), wavelet hidden Markov model (WHMM), and Block‐Matching and 3D filtering (BM3D). Lower panel: ground truth and results from deep learning methods including DnCNN, SwinIR, HoloDenoiser, Noise2Noise, Noise2Void, and Restormer. Insets show magnified views of the boxed regions for detailed comparison of fringe preservation and noise suppression.

To quantitatively evaluate denoising performance, we simulated holograms from a *p–n* junction as test data. In our simulation, phase changes linearly from −1 to 1 radian, with a weak gradient of 0.05 radian/pixel at the junction. We then compare HoloDenoiser with conventional methods widely adopted in this field (BF: bilateral filtering, MF: median filtering, TVD: total variation denoising, WF: Wiener filtering, and WHMM: wavelet hidden Markov model, BM3D: Block‐Matching and 3D filtering). Furthermore, we evaluate it against state‐of‐the‐art deep learning models. Due to the lack of deep learning frameworks specifically designed for off‐axis electron holography denoising, we selected representative benchmark models and optimally adapted them for this study (DnCNN [47], Noise2Noise [30], Noise2Void [32], SwinIR [48], and Restormer [49]). It is seen that there are characteristic limits for each approach (Figure 3). Conventional methods exhibit trade‐offs between noise suppression and structural preservation, including edge softening, jagged boundaries, staircase artifacts, or attenuated contrast. Deep learning methods instead show variable performances, ranging from residual noise and contrast loss to excessive smoothing of fringe structures. In contrast, HoloDenoiser maintains sharp fringe edges, accurate curvature across the junction region, and optimal contrast, yielding the closest match to the ground truth in both intensity profiles and reconstructed phase (see Section S2.2 and Figure S4).

**FIGURE 4:**
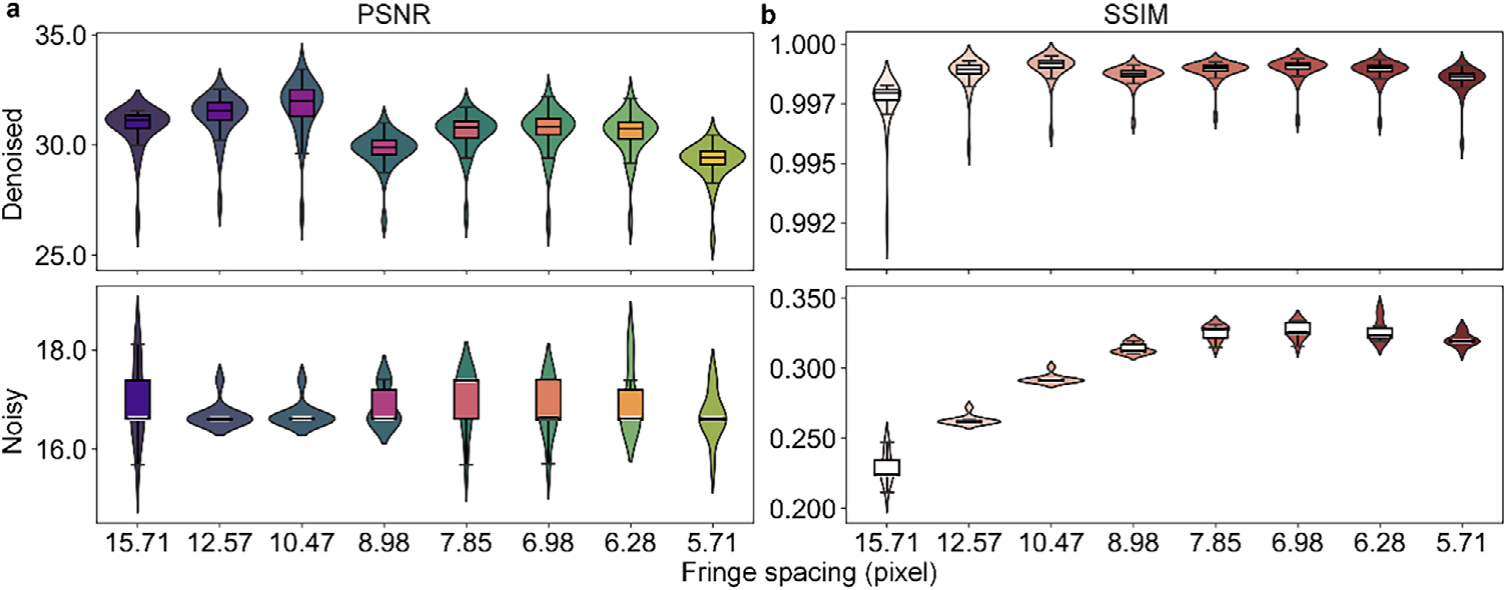
Quantitative comparison of PSNR and SSIM values before and after denoising under varying fringe spacing parameters. (a), Distributions of peak signal‐to‐noise ratio (PSNR) for denoised holograms and corresponding noisy inputs. (b), Distributions of structural similarity index (SSIM) for denoised holograms and corresponding noisy inputs. All results are evaluated at a dose of 1 e^−^/pixel.

We further confirm the robustness of HoloDenoiser on different fringe spacing parameters. As shown in Figure 4, the model achieves exceptionally higher PSNR and SSIM values than the corresponding noisy inputs across the test dataset at a low dose condition of 1 e^−^/pixel. PSNR measures the pixel‐wise fidelity between the reconstructed image and the reference image based on the mean squared error, while SSIM evaluates the structural similarity by considering luminance, contrast, and structure. Higher PSNR and SSIM values indicate better reconstruction performance. For the noisy inputs, the PSNR values remain considerably lower across the fringe spacings and drop further as the fringes become denser. The SSIM values show a less monotonic response: Initially increase as the fringe spacing decreases, but begin to fall once the spacing becomes sufficiently small, indicating a loss of structural similarity that is broadly consistent with the degradation reflected by PSNR. In contrast, the SSIM values of the denoised holograms remain consistently above 0.997, indicating that the structural fidelity of the denoised holograms is largely preserved regardless of fringe frequency. However, a gradual decline in PSNR is observed as the fringe spacing decreases from ∼15.71 to ∼5.71 pixels. This degradation can be attributed to the fact that finer fringes approach the Nyquist sampling limit, where each fringe period is represented by fewer pixels. Under such conditions, the discrete sampling of the sinusoidal fringe pattern becomes increasingly coarse, reducing the effective signal contrast encoded in the hologram and leaving less redundant spatial information for the network to exploit during denoising. In addition, we conduct ablation studies to evaluate the contribution of each module within HoloDenoiser, and the results are summarized in Table S1.

### 5.2 Evaluation on Experimental Datasets

We then evaluate the performance of HoloDenoiser using experimental electron holograms, where no specimen is present. This means the phase in the holograms should be flat or zero, acting as an ideal platform to test the noise level. In addition, we also compare the common acquisition modes of the direct electron detector, i.e., Linear and Counting, as a function of the (accumulated) electron dose.

**FIGURE 5:**
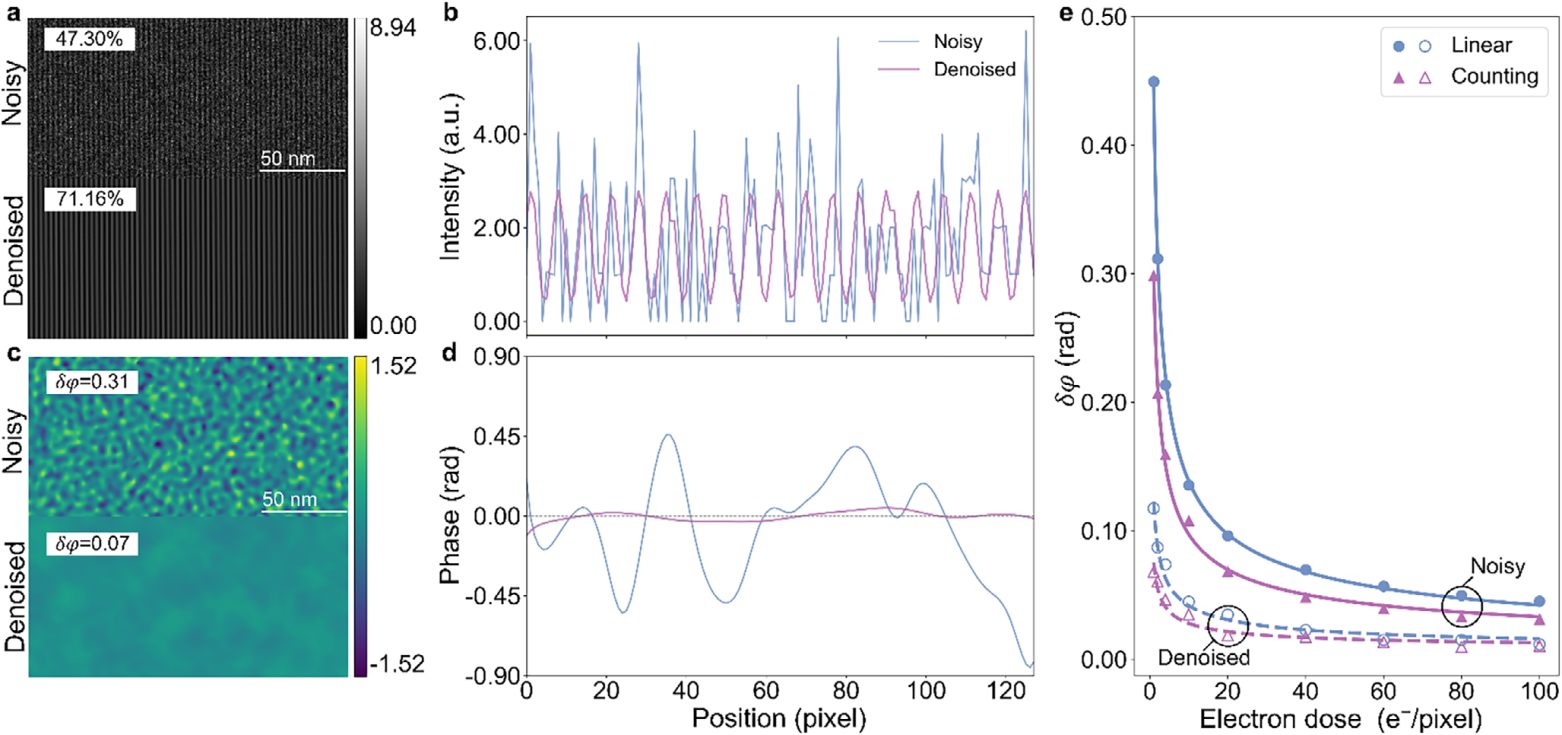
Evaluation of denoising performance using experimental holograms as a function of the electron dose. (a), Noisy hologram (upper) and denoised hologram (lower) acquired at an electron dose of 1 e^−^/pixel (pixel size = 0.36 nm) in Counting mode. (b), Central horizontal line profiles of the intensity extracted from (a), where blue and purple curves represent the noisy and denoised signals, respectively. (c), Corresponding phase images reconstructed from the above holograms shown in (a). (d) Central horizontal line profiles of the phase extracted from (c). (e) Standard deviation in the phase (δφ) as a function of the electron dose ranging from 1 to 100 e^−^/pixel, acquired in Linear (blue) and Counting (purple) modes, before (solid symbols) and after (open symbols) denoising. Corresponding fit curves to the experimental data are plotted in solid and dashed lines.

The Gatan K2 direct electron detector operates in two distinct modes—Linear and Counting—which exhibit fundamentally different noise characteristics. In Linear (integrating) mode, the detector accumulates analog charge. This process introduces electronic readout noise, modulation transfer function (MTF) broadening, and Landau noise arising from the stochastic variability of energy deposition by incident electrons, all of which degrade the detection quantum efficiency (DQE). On the other hand, the Counting mode employs a thresholding algorithm to identify individual electron events. By normalizing the energy deposition, this approach effectively eliminates both Landau and readout noise. To evaluate the generalizability of HoloDenoiser across different detector modes, separate models were trained for each mode. Both were pretrained on simulated holograms as a function of electron doses and spatial frequency of fringes. The pretrained models were then fine‐tuned on corresponding experimental holograms. Figure 5 presents the denoising performance of HoloDenoiser in experimental holograms.

At a representative electron dose of 1 e^−^/pixel (pixel size = 0.36 nm) in Counting mode, apparent noise in raw holograms (Figure 5a, upper) is suppressed after denoising (Figure 5a, lower). The contrast of fringes is increased from ∼47.30% to ∼71.16%. This effect is also confirmed by representative line profiles in the two holograms, as shown in Figure 5b. Corresponding phase images exhibit a similar trend (see Figure 5c,d). The standard deviation of the phase, δφ, measured from artifact‐corrected central regions, is improved from ∼0.31 to ∼0.07 radian. We then evaluate δφ as a function of the electron dose, ranging from 1 to 100 e^−^/pixel in both acquisition modes, see Figure 5e. In the Linear mode, δφ in raw holograms decreases from ∼0.45 to ∼0.05 radian with increasing electron dose. This 10 times decrease is anticipated as stochastic noise should follow the so‐called inverse‐square‐root law at 100 times increase in the electron dose. The Counting mode exhibits a similar behavior but with superior performance due to the advantage of direct electron counting, with δφ decreased from ∼0.31 to ∼0.03 radian. After denoising, δφ is then further reduced from ∼0.11 to ∼0.01 radian in Linear mode, and from ∼0.07 to ∼0.01 radian in Counting mode. Note that this reduction trend remains largely consistent with the inverse‐square‐root law, suggesting that the denoised phase precision is still fundamentally governed by the electron statistics. In addition, despite their different noise characteristics, both modes show a similar enhancement, with almost a factor of 3–4 reduction in δφ, irrespective of the electron dose (see Section S2.3 Validation on Experimental Vacuum Data and Figure S5).

**FIGURE 6:**
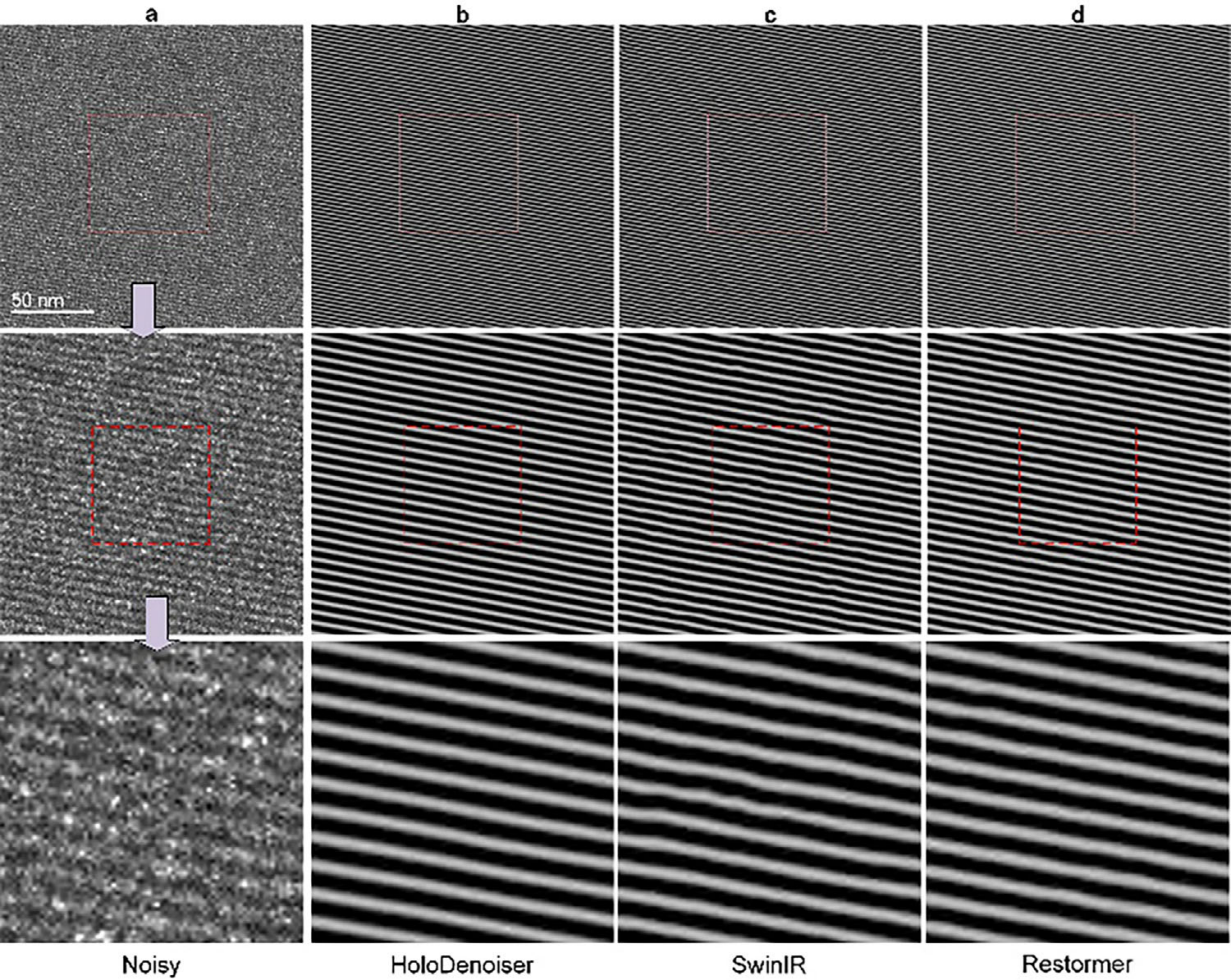
Comparison between advanced denoising models and HoloDenoiser on the Linear mode in vacuum hologram. Upper row: noisy hologram (dose = 1 e^−^/pixel, pixel size = 0.36 nm) and images processed by HoloDenoiser, SwinIR and Restormer, respectively. Lower row: magnified views of the marked regions in the upper row, showing detailed fringe structures.

The denoised data (Table 1) can be well described by the inverse‐square‐root law ($\delta \varphi =C/\sqrt{N}$, where *C* is a fitting parameter and *N* is the average electron count per pixel [50, 51]) across all conditions. This further confirms that denoising preserves rather than distorts the functional form of the dose‐noise relationship. The fitted *C* values further reveal that denoising reduces the noise by factors of ∼6.2, ∼3.8, and ∼3.5 for simulated Counting, experimental Counting, and experimental Linear modes, respectively. Furthermore, we compare the denoising performance with two other reported methods—Restormer and SwinIR (see Figure 6). Only HoloDenoiser effectively preserves fringe structure integrity after denoising, demonstrating superior generalization capability and robustness on experimental data.

**TABLE 1:** Fitting parameters for the scaling of phase standard deviation across different camera modes. The standard deviation of the phase (δφ) was fitted to the inverse square root law $\delta \varphi =C/\sqrt{N}$ for each mode and condition. C is a prefactor; R^2^ is the coefficient of determination of the fit.

Mode	Condition	*C*	R^2^	Fold‐reduction in *C*
Simulation – Counting	Noisy	0.1954	0.9988	6.2↓
	Denoised	0.0316	0.9644	
Experimental – Linear	Noisy	0.4424	0.9991	3.5↓
	Denoised	0.1251	0.9765	
Experimental – Counting	Noisy	0.3020	0.9960	3.8↓
	Denoised	0.0792	0.9602	

**FIGURE 7:**
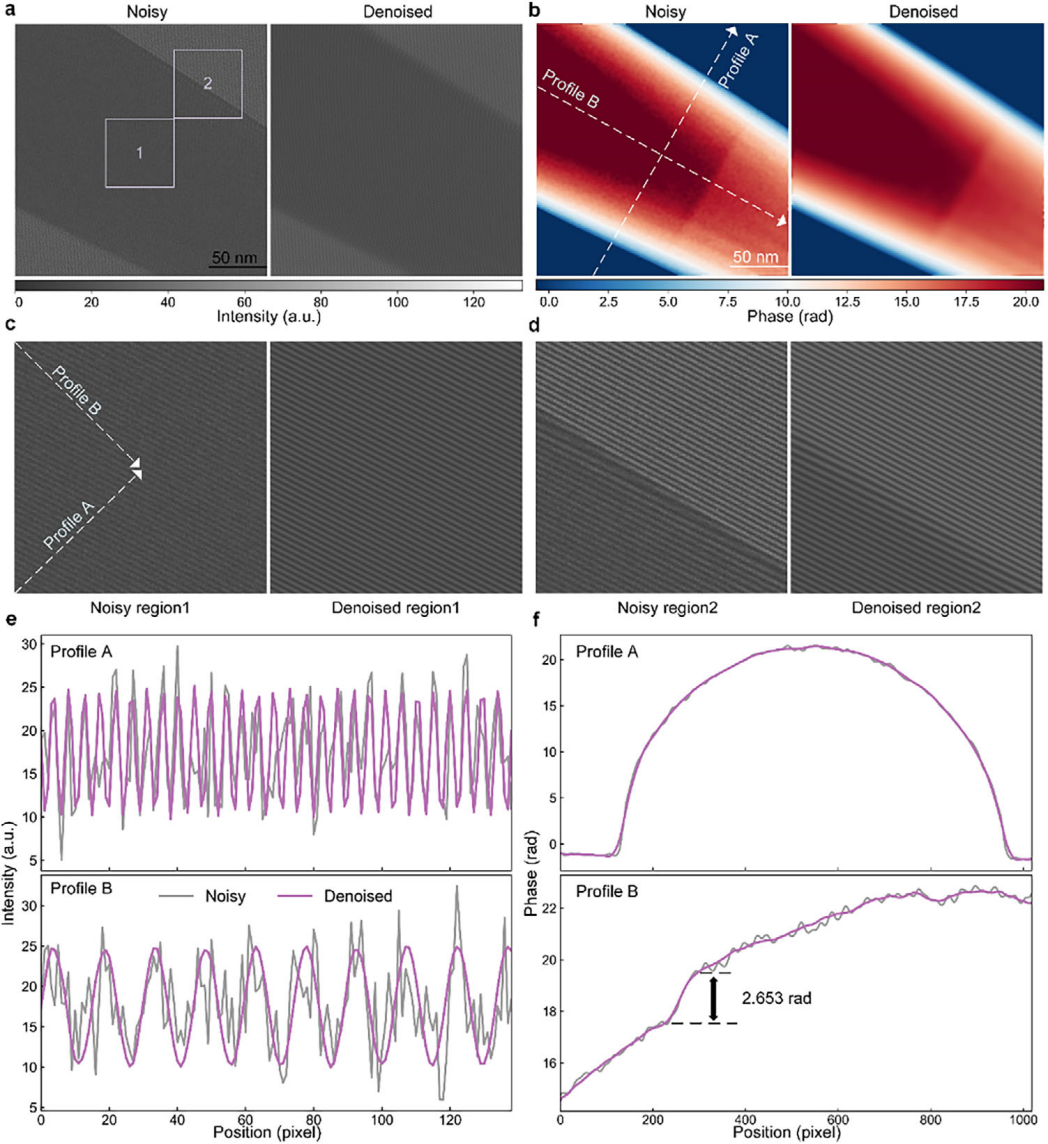
Denoising results of experimental GaAs *p–n* junction hologram. (a) Noisy and denoised holograms (dose = 30 e^−^/pixel, pixel size = 0.21 nm). (b) Reconstructed phase images. (c,d), Magnified views of the boxed regions 1 and 2 in (a), corresponding to the specimen interior and specimen‐vacuum boundary, respectively. (e) Intensity line profiles extracted along Profile A and Profile B directions from (c). (f) Phase line profiles from (b). In (e) and (f), gray and purple curves correspond to the noisy and denoised results, respectively.

We then apply HoloDenoiser to experimental holograms from a GaAs *p–n* junction in order to measure the built‐in potential (see Figure 7). No region‐specific fine‐tuning was performed for the GaAs *p–n* junction hologram. The model trained on experimental vacuum holograms was applied directly to the hologram. Notably, while the model was trained exclusively on 512 × 512 pixels patches, the experimental holograms used here are of substantially larger dimensions, demonstrating that the patch‐based training strategy successfully generalizes to high‐resolution images without retraining. After denoising, intensity fluctuations in the noisy hologram are substantially suppressed while the fringe structure remains intact (Figure 7a). Areas 1 and 2 in Figure 7 are magnified regions selected to visualize denoising performance in the specimen interior and at the specimen‐vacuum boundary, respectively (Figure 7c,d). In the specimen region, the model achieves effective noise suppression while preserving smooth intensity variations; at the boundary, fringe contrast is well maintained, indicating that the denoising process does not substantially alter the original intensity distribution. Intensity line profiles taken along the diagonal of Area 1 (Figure 7c) show that the denoised results recover the sinusoidal fringe modulation from noisy data (Figure 7e), and phase line profiles reveal a clean phase step of ∼2.653 rad across the junction corresponding to the built‐in potential (Figure 7f). Taken together, these experimental results demonstrate that HoloDenoiser effectively suppresses noise while faithfully preserving intensity distributions, and phase accuracy under practical imaging conditions. This capability enables a substantial reduction in the required electron dose without compromising phase precision, which is particularly important for beam‐sensitive materials and biological samples.

### 5.3 Pushing Phase Detection Limits at a Low Electron Dose

The ultimate aim for hologram denoising methods is the minimum phase that can be reliably recovered using as low an electron dose as possible, in order to address samples with tiny phase and/or susceptible to beam damage. Here we take a representative example of a magnetic dipole in a nano‐disk. To establish quantitative performance boundaries in this challenging regime, we conducted systematic simulations using two canonical magnetic configurations that span the range of field topologies encountered in experimental studies: a homogeneously magnetized disk and a magnetic vortex disk [52, 53]. Here, the magnitude of the magnetic dipole can be tuned to control the phase shift; when the magnitude equals one µB, it can be regarded as a single spin. The homogeneously magnetized disk produces a stray field whose Aharonov–Bohm phase decays as 1/r outside the disk, yielding an extended dipolar phase distribution. The magnetic vortex disk was described using the radial phase model (Equation S10 and Figure S2), in which the phase varies with the radial coordinate *r_m_* inside the disk and is set to zero outside the disk radius *R_m_*. This configuration features nearly closed flux lines, confining the phase signal predominantly within the disk and its vortex core.

In off‐axis holography, spatial resolution is set by the aperture, which discards every spatial frequency outside it at reconstruction. Since HoloDenoiser works on the hologram, denoising leaves this aperture untouched and cannot change the resolution it imposes. What denoising does is raise the SNR within that fixed passband. We make this trade‐off visible for a magnetic vortex disk by reconstructing at two aperture radii, *q_c_* /3 and *q_c_* /2, and placing the noisy and denoised phase side by side (see Section S2.4 Denoising Performance for Weak Magnetic Phases, and Figure S6). A wider aperture recovers finer detail but lifts the background of the noisy reconstruction, while the denoised reconstruction stays clean at both radii. With the reconstruction bandwidth fixed at *q_c_* /3, we turn to studying the phase detection limit.

**FIGURE 8:**
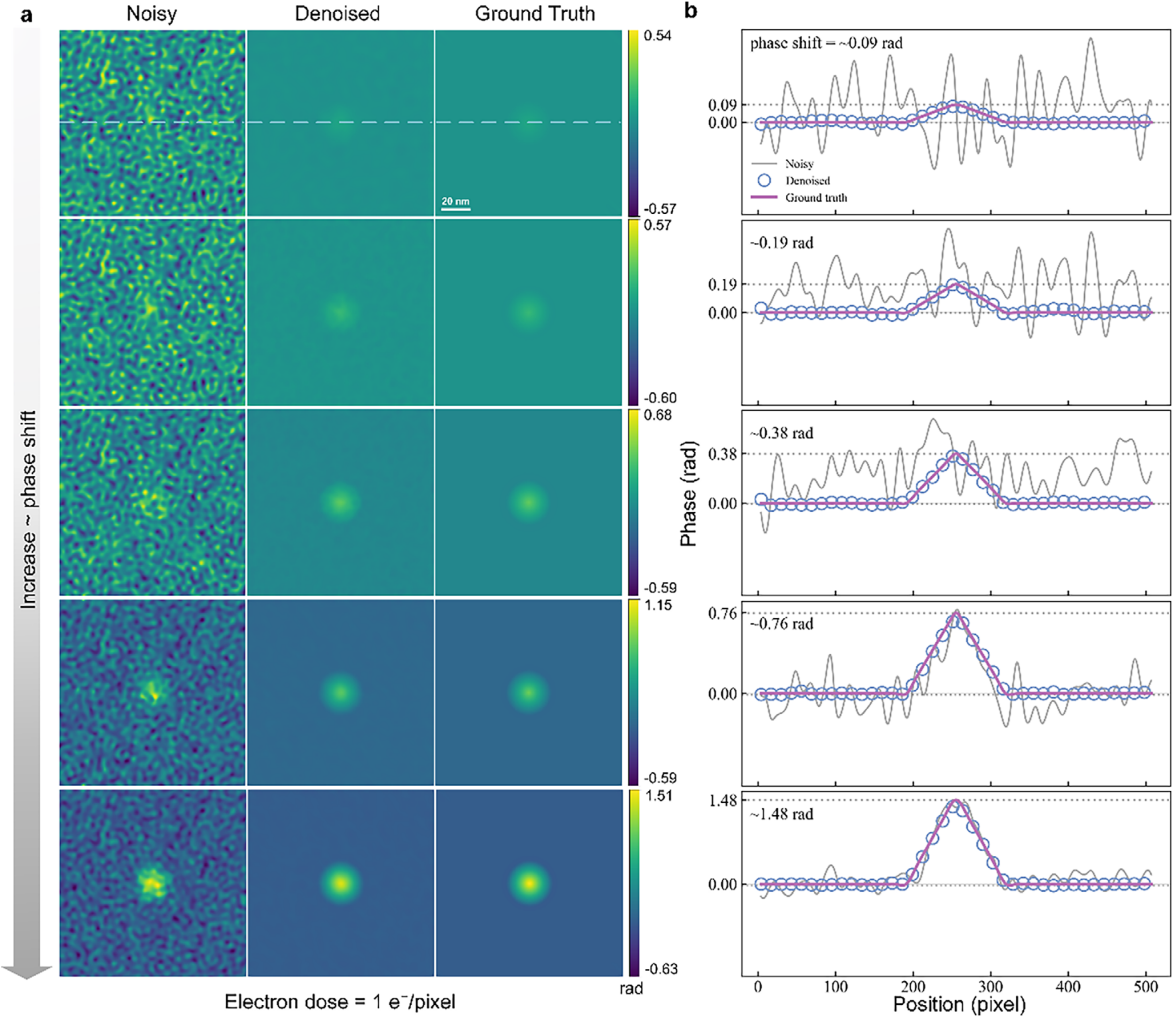
Denoising and reconstruction in magnetic vortex disk under different phase shift. (a) Reconstructed phase maps. The dose is fixed at 1 e^−^/pixel, and the phase shift increases from ∼0.09 to ∼1.56 rad from top to bottom. For each row, the leftmost column displays the raw noisy phase map, while the subsequent columns show the HoloDenoiser‐denoised phase maps and ground truth maps, respectively. (b) One‐dimensional phase profiles extracted along the dashed line in (a). The gray curves denote noisy reconstructions, the red dashed curves show the ground truth, and the blue curves with circle markers correspond to the denoised outputs.

**FIGURE 9:**
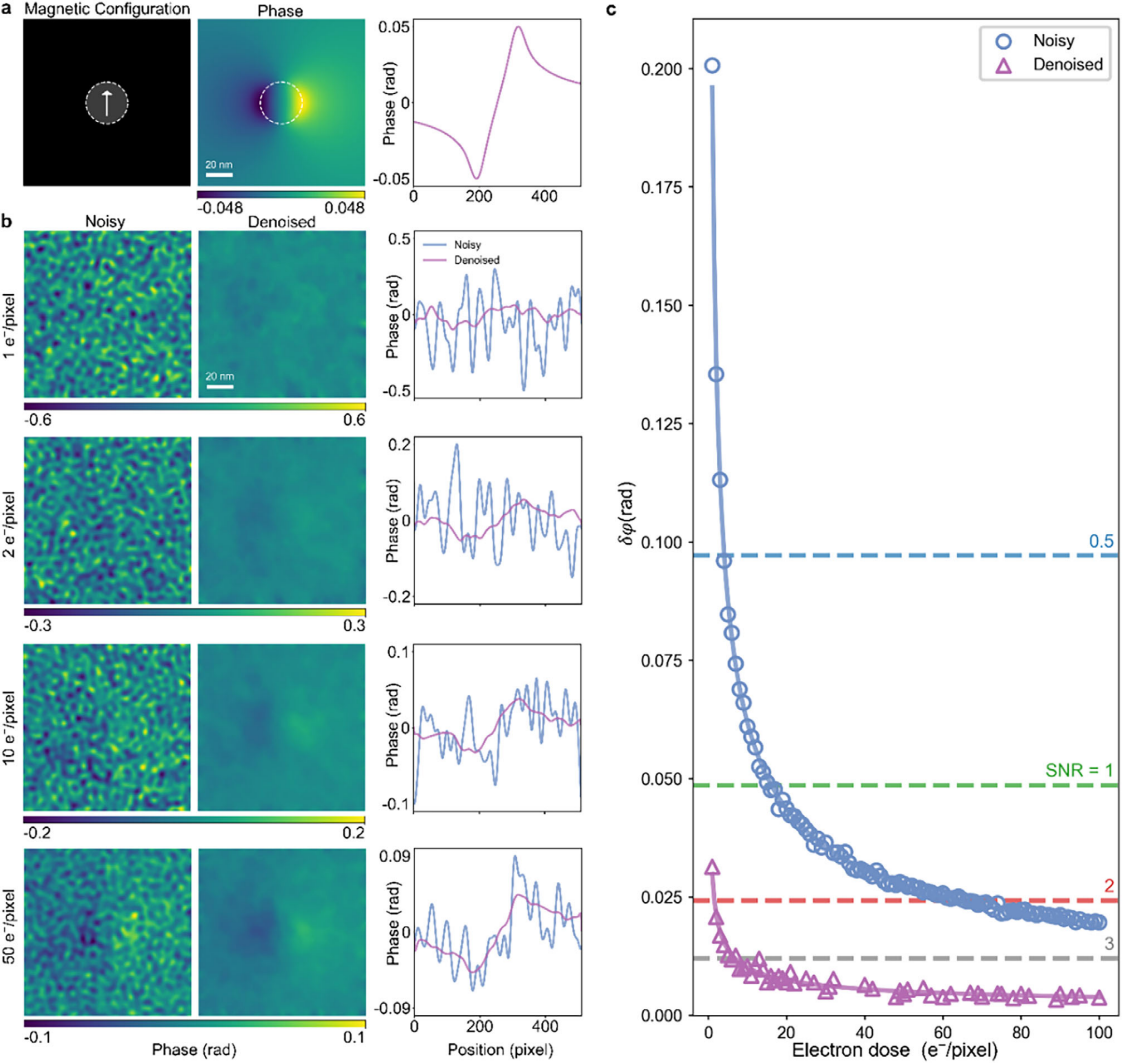
Pushing phase detection limit at a low electron dose. (a) Magnetic configuration of a homogeneously magnetized disk (left), corresponding phase map (middle) with a maximum phase shift of ∼0.048 radian, and central horizontal phase profile (right). (b) Noisy (left) and denoised (middle) phase images, and corresponding central horizontal phase profiles (right) at a fixed phase shift with increasing electron doses of 1, 2, 10, and 50 e^−^/pixel (top to bottom). (c) Phase standard deviation (δφ) as a function of electron dose (1–100 e^−^/pixel), before (blue circles) and after (purple triangles) denoising. Dashed lines indicate SNR levels based on the expected phase of ∼0.05 radian. Solid lines represent fit curves to the data.

We first examined the phase detection limit as a function of phase magnitude at a fixed low dose of 1 e^−^/pixel. For magnetic vortex configurations, characteristic bipolar magnetic phase patterns were successfully recovered from ∼0.09 radian to ∼1.56 radian (Figure 8). At this condition, the raw phase maps are dominated by shot noise, rendering the vortex‐induced bipolar phase feature barely discernible, particularly at small phase shifts (Figure 8a, left column). After processing with HoloDenoiser, the bipolar magnetic phase distribution is effectively recovered across all tested conditions, in close agreement with the ground truth (Figure 8a, middle and right columns). One‐dimensional phase profiles extracted along the dashed line in Figure 8a further confirm that the denoised results agree well with the ground truth (Figure 8b). Similarly, circular phase spots from homogeneously magnetized disks—barely discernible in raw images—were clearly reconstructed (see Section S2.4 and Figure S7). We then pushed the phase shift toward even lower values to probe the detection limit. The weakest signal of ∼0.048 radian could not be recovered: at 1 e^−^/pixel, the phase noise before denoising (δφ ≈ 0.201 radian) is approximately four times this signal (SNR ≈ 0.25), and no magnetic structure is discernible even after denoising (see Figure 9b, top row).

To further probe this lower bound, we fixed the maximum phase shift at this value using the homogeneously magnetized nano‐disk model (Section S1.5 and Equation S9). In this model, the disk radius*R_m_*, thickness *T_m_*, saturation magnetic induction *B_sat_*, and in‐plane magnetization angle β determine the magnetic phase distribution. For the weak‐phase case in Figure 9, we fixed *R_m_* = 16 nm and *T_m_* = 4 nm, respectively, and the magnetic moment was set to 2.76 × 10^5^ µB, yielding a maximum phase shift of approximately 0.048 rad. We then examined the phase detection limit as a function of electron dose.

When the electron dose is further increased to 2 e^−^/pixel, it is estimated that SNR is approximately 0.5. Although the raw phase map remains visually indistinguishable from noise, the characteristic bipolar phase pattern of the magnetic dipole is successfully revealed after denoising (see Figure 9b, second row). This is further confirmed by representative line profiles across the central axis of the disk. At a higher electron dose, i.e., 10 e^−^/pixel (SNR ≈ 1), the raw phase map still shows no clear sign of a magnetic dipole feature, whereas after denoising the phase image exhibits a well‐defined dipolar pattern (see Figure 9b, third row). Corresponding line profiles exhibit typical peaks with improved accuracy compared to the case at an electron dose of 2 e^−^/pixel. At a much higher electron dose of 50 e^−^/pixel (SNR ≈ 2), the magnetic disk becomes faintly visible even in raw data, and denoising yields a phase distribution closely approximating the ideal magnetic dipole pattern with sharp boundaries (see Figure 9b, bottom row). Figure 9c shows the δφ as a function of the electron dose, ranging from 1 to 100 e^−^/pixel in both noisy and denoised results. The δφ in the raw noisy holograms decreases from ∼0.201 radian to ∼0.019 radian as the electron dose increases; after denoising, δφ is reduced to ∼0.031 radian and ∼0.004 radian at the corresponding doses. Both curves follow the inverse‐square‐root law. Notably, the Rose criterion (SNR ≈ 2–5), commonly regarded as the threshold for reliable signal discrimination, requires an electron dose of at least ∼50 e^−^/pixel to resolve a ∼0.048 radian phase without denoising, as indicated by the dashed horizontal lines (SNR = 2). With HoloDenoiser the same magnetic structure is already recovered at just 2 e^−^/pixel—a ∼25‐fold reduction in the required electron dose. This demonstrates that HoloDenoiser substantially pushes the phase detection limit into the ∼0.05 radian regime at low dose, opening possibilities for imaging beam‐sensitive specimens that were previously inaccessible.

### 5.4 Quantitative Power Spectral Density Analyses

**FIGURE 10:**
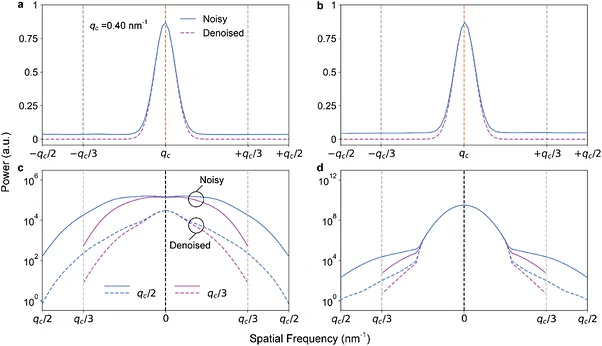
Radially averaged power spectral density (PSD) analysis of simulated and experimental vacuum holograms acquired in Counting mode at an electron dose of approximately 1 e^−^/pixel. Orange dashed lines mark *q_c_* (denotes the carrier frequency), gray dashed lines indicate *q_c_* /3 and *q_c_* /2. (a) PSD of the simulated vacuum hologram before (blue solid) and after (purple dashed) denoising. (b) PSD of the experimental vacuum hologram before and after denoising. (c) PSD of the reconstructed phase maps from the simulated vacuum hologram, shown on a logarithmic scale, where the positive and negative halves correspond to phase maps reconstructed from the positive (+*q_c_*) and negative (‐*q_c_*) sidebands, respectively. Two reconstruction aperture sizes are compared: *q_c_* /2 (solid lines) and *q_c_* /3(dashed lines). (d), PSD of the reconstructed phase maps from the experimental vacuum hologram on a logarithmic scale, with the same settings as in (c).

In order to reveal which information is removed or suppressed via the neural network, we compute the radially averaged power spectral density (PSD) of holograms and reconstructed phase maps before and after denoising in simulated and experimental holograms. In the PSD of the simulated hologram (Figure 10a), it is seen that around the carrier frequency (*q_c_*), information is well preserved, showing no measurable change both in the peak and the width before and after denoising. Instead, away from *q_c_*, it is obvious that the intensity of the power spectrum is heavily suppressed, indicating the neural network effectively identifies and suppresses noise superimposed on the interference fringes rather than indiscriminately indiscernibly smoothing the entire image. A similar behavior is also observed in experimental holograms (Figure 10b).

The denoised phase map from simulated holograms exhibits a uniformly lower PSD after denoising across the entire frequency range, irrespective of the aperture size (Figure 10c). For the experimental data (Figure 10d), noise suppression at mid‐to‐high frequency regimes is seen to be more effective than at a relatively lower frequency regime. This behavior is distinct from that observed in the simulated data (Figure 10c), likely because the simulations do not include low‐frequency contributions such as instrumental instabilities and slowly varying background signals. The network relies on the statistical independence of noise between paired observations; identical features across multiple acquisitions are retained rather than suppressed. This could be tackled by network training with time series experimental data.

## 6 Conclusions

In this paper, we present HoloDenoiser, a deep neural network dedicated to denoising electron holograms by exploiting their characteristic spectral structure, using off‐axis electron holography as the demonstration benchmark. Experiments on both simulated and experimental holograms demonstrate that HoloDenoiser reduces phase noise by a factor of 3–4 while faithfully preserving the inverse‐square‐root scaling law, outperforming conventional denoising techniques and state‐of‐the‐art deep learning models. For weak phase objects (∼0.05 radian), characteristic phase patterns are recovered with an approximately 25‐fold reduction in electron dose, opening new possibilities for imaging beam‐sensitive materials and biological specimens. Quantitative power spectral density analyses further confirm that the network selectively suppresses noise across the spectrum while leaving the carrier frequency peaks and sideband structure intact, providing frequency‐resolved evidence of faithful phase preservation.

HoloDenoiser performs reliably across the data examined here, yet its operating range has limits. The proposed method relies on a spectral prior that locates the sideband in the Fourier space and isolates it with Gaussian masks. Two situations undermine this prior. At very low dose, the sideband peak barely thresholds the noise floor. In addition, when biprism artifacts or Fresnel fringes introduce spurious peaks in the spectrum, the search ring can lock onto the wrong sideband position. For experimental training, we can therefore introduce the prior selectively, according to the image quality. A second limit comes from the denoising principle: Noise is removed only when it is independent between paired observations, so correlated noise that repeats across frames, such as fixed‐pattern noise and detector artifacts, is retained rather than suppressed. Furthermore, holograms whose carrier frequency or phase variation is distinct markedly from the training conditions would call for retraining or fine‐tuning.

Even in its present form, the design principles may transfer broadly to interferometric modalities beyond electron holography, including optical and X‐ray holography. We believe HoloDenoiser establishes a strong baseline for deep‐learning holographic denoising and provides a generalizable framework for noise‐limited interferometric measurements.

## Author Contributions

**Ye Luo**: conceptualization, validation, writing – original draft, methodology, data curation, writing – review and editing. **Luyang Wang**: data curation, validation. **Yu Han**: writing – review and editing. **Thibaud Denneulin**: writing – review and editing, resources. **Zhiling Yu**: validation, methodology. **Xiaoyu Wang**: methodology, validation. **Qi Liu**: writing – review and editing, resources, project administration. **Fengshan Zheng**: writing – review and editing, resources. **Xiaowen Chen**: methodology, validation. **Ya‐Hui Jia**: writing – review and editing. **Rafal E. Dunin‐Borkowski**: writing – review and editing.

## Conflicts of Interest

The authors declare no conflicts of interest.

## Supporting information




**Supporting File**: advs77380‐sup‐0001‐SuppMat.docx.

## Data Availability

The training code and datasets that support the findings of this study are available in the GitHub repository at https://github.com/YLLY‐6/HoloDenoiser.
