# Supplementary material for: Deep‐Learning‐Based Denoising for Improved Phase Precision in Electron Holography of Electromagnetic Fields in Nanoscale Materials
Source: Adv Sci (Weinh). 2026 Aug 31:e77380. Online ahead of print. doi: 10.1002/advs.77380 (PMC13528685; doi:10.1002/advs.77380)
Supplement: Supplementary file 1 [file ADVS-9999-e77380-s001.docx]

Supplementary Information

Ye Luo^1^, Luyang Wang^2,3^, Zhiling Yu^2,4^, Xiaowen Chen^2,3^, Xiaoyu Wang^2,3^, Ya-Hui Jia^1^, Thibaud Denneulin*^5^, Yu Han^2,4^, Rafal E Dunin-Borkowski^5^, Fengshan Zheng*^2,3^ and Qi Liu*^1^

^1^School of Future Technology, South China University of Technology, Guangzhou, China. ^2^Center for Electron Microscopy, South China University of Technology, Guangzhou, China. ^3^Spin-X Institute, School of Physics and Optoelectronics, Guangdong-Hong Kong-Macao Joint Laboratory of Optoelectronic and Magnetic Functional Materials, South China University of Technology, Guangzhou, China. ^4^School of Emergent Soft Matter, South China University of Technology, Guangzhou, China. ^5^Ernst Ruska-Centre for Microscopy and Spectroscopy with Electrons, Forschungszentrum Jülich, Jülich, Germany.

*Corresponding author: [t.denneulin@fz-juelich.de](mailto:t.denneulin@fz-juelich.de) (T. Denneulin); [zhengfs@scut.edu.cn](mailto:zhengfs@scut.edu.cn) (F. Zheng); [drliuqi@scut.edu.cn](mailto:drliuqi@scut.edu.cn) (Q. Liu)

1. Supplementary methods

This supplementary section provides comprehensive descriptions of the baseline methods employed for comparative evaluation. Given that the primary contribution of this work lies in demonstrating the efficacy of deep learning for electron hologram denoising, our comparative analysis is structured around two principal objectives: (1) to establish the superiority of deep learning methods over conventional signal processing techniques, and (2) to evaluate the advantages of the proposed architecture relative to representative deep learning baselines. For traditional methods, six approaches representing distinct methodological paradigms were selected: spatial-domain filtering (median filter and bilateral filter), frequency-domain filtering (Wiener filter), variational methods (total variation denoising), statistical modeling (wavelet hidden Markov model), and Block-Matching and 3D filtering (BM3D) ^1-3^. For deep learning methods, five representative architectures were chosen: DnCNN ^4^ Noise2Noise ^5^, Noise2Void ^6^, SwinIR ^7^, and Restormer ^8^.

**1.1. Traditional Denoising Methods**

Conventional approaches to image restoration rely on analytical signal processing with explicit mathematical priors. We evaluated five methods representing distinct paradigms:

1) **Local spatial filtering methods**

These methods, including median filter (MF) and bilateral filter (BF), operate on pixel neighborhoods to suppress noise while preserving edges. The median filter replaces each pixel value with the median of its surrounding neighborhood, providing robust suppression of impulse-type noise without introducing blurring at edge boundaries. The bilateral filter extends this concept by jointly weighting neighboring pixels according to both their spatial distance and intensity similarity, thereby achieving adaptive smoothing that preserves significant intensity transitions while averaging out random fluctuations in homogeneous regions.

2) **The Wiener filter (WF)**

WF provides an optimal linear estimate in the frequency domain by weighting spectral components according to estimated signal-to-noise ratios. It is derived from the minimization of the mean squared error between the estimated and true signals, yielding a transfer function that attenuates frequency components dominated by noise while passing those with high signal energy.

3) **Total variation denoising (TVD)**

TVD minimizes an energy functional combining data fidelity with gradient sparsity regularization, promoting piecewise-smooth solutions that effectively reduce noise while maintaining sharp edges. The total variation regularization term penalizes the integral of the gradient magnitude across the image, encouraging solutions with sparse gradients and thus preserving discontinuities such as edges and boundaries. The balance between data fidelity and regularization is controlled by a weighting parameter, allowing flexible adjustment of the trade-off between noise suppression and detail retention across varying noise levels.

4) **Wavelet hidden Markov model (WHMM)**

WHMM exploits statistical dependencies among wavelet coefficients across scales and orientations through a hidden Markov tree structure. In this framework, each wavelet coefficient is associated with a hidden state that captures whether the coefficient corresponds to a significant signal feature or to noise, and the transition probabilities between parent and child nodes encode the persistence of important structures across decomposition levels. By performing Bayesian estimation conditioned on these learned inter-scale priors, WHMM achieves superior noise suppression compared to simple wavelet thresholding while better preserving multi-scale structural details.

5）**Block-Matching and 3D filtering (BM3D)**

BM3D exploits the nonlocal self-similarity of images by searching for mutually similar 2D patches across the image and stacking them into 3D arrays, a process known as block-matching and grouping. Collaborative filtering is then performed in the 3D transform domain, where a three-dimensional linear transform is applied to each group, followed by coefficient shrinkage to attenuate noise components, and an inverse transform to recover the filtered patches. This procedure is executed in two successive stages: the first stage produces a basic estimate through hard thresholding in the transform domain, while the second stage refines this estimate by applying empirical Wiener filtering using the basic estimate as a pilot signal.

Despite their diverse mechanisms, traditional methods share common limitations for electron hologram denoising: they rely on hand-crafted priors that may not capture the complex characteristics of holographic interference patterns, and lack adaptive, data-driven feature extraction capabilities.

**1.2. Deep Learning Methods**

The paradigm shift toward deep learning has introduced data-driven architectures capable of capturing complex non-linear feature representations directly from image distributions, by mitigating the limitations of hand-crafted priors. We compared against five representative architectures.

1) **DnCNN**

DnCNN is a seminal work in deep learning-based image denoising, employs stacked convolutional layers with batch normalization and residual learning to estimate the noise component directly. Its architectural simplicity and well-established performance make it an essential baseline for evaluating more sophisticated approaches.

2) **Noise2Noise/Noise2Void**

Noise2Noise employs a U-Net ^9^ backbone with symmetric encoder–decoder structure and skip connections. Its key innovation lies in the self-supervised training paradigm, wherein the network learns from pairs of independently corrupted observations without requiring clean ground-truth images. Noise2Void further relaxes the data requirements by eliminating the need for paired noisy images. While it also typically utilizes a U-Net backbone, its core innovation is the blind-spot network strategy, which enables self-supervised learning from single noisy images. By systematically masking specific pixels (blind spots) during training and forcing the network to predict these masked values using only their spatial neighborhoods, Noise2Void prevents the network from learning a trivial identity mapping.

3) **SwinIR and Restormer**

SwinIR and Restormer represent significant advancements in applying Vision Transformers to image restoration. Both models adopt U-Net-style encoder–decoder architectures augmented with sophisticated attention mechanisms to overcome the quadratic computational complexity of standard self-attention when processing high-resolution images. SwinIR leverages Residual Swin Transformer Blocks (RSTB), utilizing a shifted window-based self-attention mechanism. Restormer introduces a Multi-Dconv Head Transposed Attention (MDTA) module, which computes self-attention across the channel dimension rather than the spatial dimension, drastically reducing memory and computational overhead. Furthermore, Restormer incorporates depth-wise convolutions within its feed-forward network to explicitly encode spatially local context. This dual strategy allows Restormer to efficiently aggregate global channel-wise representations while preserving fine-grained spatial details, making it highly adept at computationally demanding restoration tasks.

These architectures share a critical limitation for electron hologram processing: standard downsampling operations, e.g., strided convolutions or pooling, inherently violate the Nyquist sampling criterion, leading to spectral aliasing. We address this by incorporating anti-aliasing modules in both downsampling and upsampling pathways^10^^,^ ^11^.

**1.3.** **HoloDenoiser architecture**

HoloDenoiser is an encoder–decoder network designed for electron hologram denoising (Fig. SM1). The architecture incorporates several key components addressing challenges to holograms:

1) **Fast Fourier convolution (FFC) blocks**

Standard convolutions operate locally with fixed-scale kernels, limiting their ability to capture the extended periodic structures inherent to holographic fringes. We adopted the Fast Fourier Convolution (FFC) framework proposed by Chi et al., which provides non-local receptive fields within a single convolutional unit ^12^. According to the spectral convolution theorem, point-wise updates in the frequency domain globally affect all input features, enabling efficient global context modeling without the quadratic complexity of self-attention. The FFC block processes features through two interconnected pathways. The local branch applies standard convolutions for local feature extraction with translation equivariance. The global branch employs a Fourier Unit that transforms features to the frequency domain via 2D FFT, applies learnable convolutions, then transforms back via inverse FFT. Cross-connections between branches facilitate bidirectional information exchange through four pathways: local-to-local, local-to-global, global-to-local, and global-to-global, all implemented as pointwise convolutions. This enables the network to learn complementary spatial-frequency representations for capturing both fine-grained fringe textures and global periodic coherence.

2) ***Anti-aliasing* processing**

*Aliasing* presents a central challenge when carrier fringes contain spatial frequencies approaching the Nyquist limit. Direct downsampling folds high-frequency content into lower bands, introducing irreversible artifacts that distort sideband peaks and elevate phase noise. HoloDenoiser adopts a frequency-aware dual-pathway *anti-aliasing* strategy. The spatial pathway pre-filters feature with a depthwise low-pass blur before strided convolution. In parallel, a frequency-domain pathway performs Fourier-domain low-pass filtering using a radially defined mask with smooth transition bands. The two filtered signals are combined via learnable fusion weights, enabling adaptive balance depending on fringe characteristics. During upsampling, features are resized by bilinear interpolation with optional smoothing to reduce interpolation artifacts.

3) **Window attention**

At the bottleneck layer, the feature map is compressed to a minimum spatial extent. Distinguishing between weak spectral features in the sideband regions as weak sample signals to be retained or residual noise to be suppressed requires comparison with the surrounding spatial context. Meanwhile, fringe patterns extend smoothly across the entire field of view, and maintaining this continuity is essential for accurate phase reconstruction. When partitioning features into local windows, boundary discontinuities can arise. The overlapping window attention module addresses this through 50% overlapping windows combined with Gaussian-weighted reconstruction, where each pixel receives contributions from multiple windows blended using weights that decay smoothly from window centers. Information exchange between windows is achieved through alternating shifted windowing in deeper stages:

$$\begin{aligned} G_{i}\left( p \right)= \exp\left( -\frac{{x_{i}}^{2} + {y_{i}}^{2}}{2\alpha^{2}} \right)\#\left( \mathrm{SM}1 \right) \end{aligned}$$

$$\begin{aligned} X_{out}\left( p \right)= \frac{\sum_{i} G_{i}\left( p \right)\cdot O_{i}\left( p \right)}{\sum_{i} G_{i}\left( p \right)}\#(SM2) \end{aligned}$$

Where $p$ is any spatial location that receives contributions from multiple overlapping windows; $O_{i}\left( p \right)$ is the self-attention output of the *i*-th window at position $p$; $G_{i}\left( p \right)$ is a two-dimensional Gaussian weighted kernel with the geometric center of window *i* as the origin; $x_{i}$, $y_{i}$ are the coordinates of $p$ relative to the center of window. Each attention block is followed by a feed-forward network. We adopt the Gated-Dconv Feed-Forward Network (GDFN) from Restormer ^8^, which replaces the standard two-layer MLP with a gated structure: the input is projected to an expanded channel dimension via a pointwise convolution, processed by a shared depth-wise convolution, then split into two halves along the channel axis—one activated by GELU and element-wise multiplied with the other as a linear gate.

**Fig. SM1. HoloDenoiser Architecture.** **a**, Denoising process. The network follows an encoder-bottleneck-decoder structure with four stages at each resolution level. The input processing branch (left) automatically detects sideband positions and generates Gaussian masks as spectral priors. Spatial and frequency features are extracted in parallel and fused before entering the encoder. The encoder employs *anti-aliasing* downsampling combined with Fast Fourier Convolution (FFC) blocks. The bottleneck uses window attention with alternating shift operations. The decoder performs upsampling with fusion blocks that selectively incorporate encoder features via cross-attention, followed by a frequency protect module that preserves sideband integrity. **b**, Model components. **Left:** *anti-aliasing* downsampling module combining spatial blur and frequency-domain low-pass filtering with learnable fusion weights. **Center:** FFC block showing the local branch (spatial convolutions) and global branch (Fourier Unit), connected through four cross-connection pathways (L-L, L-G, G-L, G-G). **Bottom:** fusion block implementing selective cross-attention between encoder skip connections and decoder features through spatial gating. **Right:** window attention module with 50% overlapping windows, multi-head self-attention, Gaussian-weighted reconstruction, and gated feed-forward network (GDFN).

4) **Selective cross-attention fusion (Fusion block)**

Skip connections provide fine-grained spatial information to the decoder, but risk transmitting noise that has already been suppressed at deeper layers. The decoder addresses this by employing selective cross-attention fusion. To disambiguate signal-rich regions from noise-dominated areas, we first generate a spatial gate $G$ by leveraging the deep contextual information from the decoder feature $F_{dec}$ to filter the encoder feature $F_{enc}$:

$$\begin{aligned} G=\sigma\left( {Conv}_{1\times1}\left( ReLU\left( {Conv}_{1\times1}\left[ F_{dec},F_{enc} \right] \right) \right) \right)\#\left( \mathrm{SM}3 \right) \end{aligned}$$

$$\begin{aligned} F_{enc}^{'} = F_{enc} \odot G\#\left( \mathrm{SM}4 \right) \end{aligned}$$

The upsampled decoder features and the corresponding encoder features are concatenated to create a unified representation that captures both deep semantic context and shallow spatial textures. This combined feature map is passed through a bottleneck structure, utilizing 1x1 convolutions and non-linear activation to compress the information and extract salient spatial cues. $\sigma$ is a Sigmoid function applied to generate a pixel-wise attention map, which acts as a "soft mask." In the initial decoding phase, where the semantic gap between encoder and decoder is most pronounced, we employ a cross-attention mechanism to selectively retrieve fine-grained details, Eq. (SM5).

$$\begin{aligned} Q = W_{q}{Pool}_{sq}\left( F_{dec} \right), K = W_{k}{Pool}_{sk}\left( F_{enc}^{'} \right), \\ V = W_{v}{Pool}_{sk}\left( F_{enc}^{'} \right)\#\left( \mathrm{SM}5 \right) \end{aligned}$$

The refined decoder feature is then computed via a residual-based attention mechanism, Eq. (SM6). Where $Pool$ denotes average pooling with stride *s*, and $Up$ signifies the upsampling operation to restore the original resolution. By setting $sq$ =2 and $sk$ =4, the model prioritizes the decoder’s semantic guidance while efficiently sampling from the encoder’s feature space.

$$\begin{aligned} F_{dec}^{'} = F_{dec} + Up\left( Softmax\left( \frac{QK^{T}}{\sqrt{d_{k}}} \right) \cdot V \right)\#\left( \mathrm{SM}6 \right) \end{aligned}$$

5) **Frequency protection**

The final output undergoes frequency protection to preserve critical sideband information. This module operates in the Fourier domain using complex-valued blending: both the denoised result and original input are transformed via FFT, and adaptive weights are computed based on amplitude ratios and the detected sideband mask. Rather than separately blending amplitude and phase, which can introduce discontinuities, the module performs weighted averaging directly in the complex domain. The weight is higher in sideband regions to preserve carrier frequency fidelity and lower in background regions to maximize noise suppression. This approach naturally handles phase continuity while ensuring that denoising does not inadvertently attenuate the holographic fringes essential for phase reconstruction.

6) **Mask**

We developed an adaptive algorithm to automatically detect sidebands and generate masks in the Fourier domain. The 2D FFT of the input hologram reveals the characteristic three-peak structure: a central DC component and two symmetric sidebands encoding the object phase and its conjugate. The primary sideband is identified by searching for the maximum peak within an annular region (5%–45% of image size) using a 95th-percentile adaptive threshold. The filtering radius, estimated from the peak's full width at half maximum, is constrained to 3%–18% of the minimum dimension. Three 2D Gaussian soft masks are then generated for the primary sideband, its conjugate, and the DC component, providing smooth spectral transitions that suppress leakage and ringing artifacts. This approach requires no manual carrier frequency input and adapts automatically to varying fringe spacings and orientations.

**1.4. Loss**

To train HoloDenoiser for hologram denoising while preserving frequency-domain phase information, we designed a normalized multi-component (NMC) loss function that jointly optimizes real-space fidelity and frequency-domain phase accuracy. It comprises three terms: L1 loss, L2 loss, and the sideband loss.

L1 loss (mean absolute error) provides outlier robustness and promotes sharp reconstructions; L2 loss (mean squared error) penalizes large deviations and accelerates early convergence.

Denoising must suppress the noise floor while preserving sideband peak heights, as these are directly related to fringe contrast and object-wave amplitude. To this end, we propose the sideband peak fidelity loss $L_{\mathrm{PF}}$, which operates in the Fourier domain with an adaptive, amplitude-driven weighting scheme. The carrier frequency *q_c_* is obtained by automatic detection (see the Section **Mask**). Rather than using a fixed geometric mask, we exploit the amplitude spectrum of the ground truth itself as adaptive weights. A smooth boundary mask $M_{sb}\left( q \right)$ is defined via a sigmoid function, the per-frequency weight is defined as:

$$\begin{aligned} w\left( q \right)=\left\langle\left| \mathcal{F}_{tar}\left( q \right) \right| \right\rangle_{B}\cdot M_{sb}\left( q \right)\#\left( \mathrm{SM}7 \right) \end{aligned}$$

Where $\mathcal{F}_{tar}\left( q \right)$ is the Fourier transform of the target hologram (*i.e.*, an independent noise realization paired with the input), $\left\langle\cdot\right\rangle_{B}$ denotes averaging over the batch. This scheme is essentially parameter-free: frequency components carrying strong signals automatically receive larger weights, while the spectrally flat noise floor contributes negligibly.

Since holographic phase information is encoded in the complex Fourier coefficients at the sidebands, we impose a fidelity constraint in the complex domain:

$$\begin{aligned} L_{CF}=\frac{\sum_{q} w'(q)\left| \mathcal{F}_{tar}^{'}(q)-\mathcal{F}_{tar}(q) \right|^{2}}{{\sum_{q} w'(q)\left| \mathcal{F}_{tar}(q) \right|}^{2}}\#\left( \mathrm{SM}8 \right) \end{aligned}$$

Where $\mathcal{F}_{tar}^{'}\left( q \right)$ is the Fourier transform of the network output (denoised hologram), and $w^{'}\left( q \right)$ is the normalized weight. Meanwhile, denoising networks empirically tend to over-smooth, manifesting as attenuated sideband peaks (i.e., reduced fringe contrast). To counteract this tendency, we introduce an asymmetric penalty on the amplitude ratio.

Different loss components can differ by orders of magnitude, risking gradient dominance by a single term. To address this, we adopt an adaptive normalization scheme^13^^,^ ^14^. Baseline values for each component are recorded during the first training batch and subsequently updated via exponential moving average (EMA) across epoch^15^. Each component is divided by its baseline before summation, so that all terms contribute comparably to the total gradient. The normalization uses a sigmoid-based soft function that maps the loss-to-baseline ratio into a bounded range while preserving gradient flow. The final loss is a weighted sum of the three normalized components.

**1.5. Simulation of Magnetic Disk Structures**

For weak-phase magnetic structures, we further simulated two canonical disk-shaped configurations: a homogeneously magnetized disk and a magnetic vortex disk ^16^. For a homogeneously magnetized disk with magnetization oriented at an angle $\beta$ with respect to the reference axis, the phase can be expressed in piecewise analytical form inside and outside the disk, Eq. (SM9); the corresponding magnetic configuration and phase distribution are shown in the main-text Fig. 9a.

$$\begin{aligned} \phi_{\text{homo}}\left( r_{m},\beta\right)=\left\{ \begin{matrix} \phi_{\text{in}}\left( r_{m},\beta\right)=-\frac{C}{2}\left( y_{m}cos\beta-x_{m}sin\beta\right), & r_{m}<R_{m}, \\ \phi_{\text{out}}\left( r_{m},\beta\right)=-\frac{C}{2}\frac{R_{m}^{2}}{r_{m}^{2}}\left( y_{m}cos\beta-x_{m}sin\beta\right), & r_{m}\geq R_{m}. \end{matrix} \right.\text{ }C=\frac{\pi T_{m}B_{\text{sat}}}{\Phi_{0}}\#\left( \mathrm{SM}9 \right) \end{aligned}$$

Here, $x_{m}$ and $y_{m}$ are Cartesian coordinates centered at the disk, $r_{m}$ is the radial distance from the disk center, and $R_{m}$ is the disk radius. $B_{sat}$ is the saturation magnetic induction, $T_{m}$ is the disk thickness along the electron-beam direction, and $\Phi_{0}$ is the magnetic flux quantum. These quantities determine the proportionality coefficient $C$, which sets the overall magnetic phase amplitude.

The ideal radial phase model for the magnetic vortex disk is given by Eq. (SM10):

$$\begin{aligned} \phi_{\text{vortex}}\left( r_{m} \right)=\left\{ \begin{matrix} C\left( R_{m}-r_{m} \right), & r_{m}\leq R_{m}, \\ 0, & r_{m}>R_{m}, \end{matrix} \right.\#\left( \mathrm{SM}10 \right) \end{aligned}$$

In this expression, the phase is confined within the disk radius $R_{m}$, consistent with the nearly closed magnetic flux lines of a vortex configuration. The corresponding magnetic configuration and phase distribution are shown in Fig. SM2.

**Fig. SM2. Simulated magnetic vortex disk. a**, Magnetic configuration of a magnetic vortex disk. **b**, corresponding phase map. **c**, central horizontal phase profile.

In the ideal analytical forms of Eq. (SM9) and Eq. (SM10), the phase changes abruptly at the disk boundary $r_{m} = R_{m}$. Such a hard boundary introduces slowly decaying high-frequency components in Fourier space and can cause Gibbs ringing under a finite field of view and discrete FFT sampling. At the same time, in real nanostructures the magnetization and phase do not exhibit infinitely sharp transitions within a single grid cell, and experimental imaging is inevitably limited by finite spatial resolution and coherence length. To reduce these numerical artifacts, we introduced a radial soft-edge weight $w\left( r_{m} \right)$, controlled by a smoothing width $\Delta$ (in nm) and defined in Eq. (SM11):

$$\begin{aligned} w\left( r_{m} \right)=\frac{1}{2}\left[ 1-\text{erf}\left( \frac{r_{m}-R_{m}}{\Delta} \right) \right]\#\left( \mathrm{SM}11 \right) \end{aligned}$$

Therefore, for the uniformly magnetized disk, the phase can be rewritten as Eq. (SM12):

$$\begin{aligned} \phi_{\text{homo}}^{\text{soft}}\left( r_{m},\beta\right)=w\left( r_{m} \right)\phi_{\text{in}}\left( r_{m},\beta\right)+ \\ \left[ 1-w\left( r_{m} \right) \right]\phi_{\text{out}}\left( r_{m},\beta\right)\#\left( \mathrm{SM}12 \right) \end{aligned}$$

When $r_{m} \ll R_{m}$ one has $w\left( r_{m} \right) \approx1$, and the softened phase $\varphi_{\mathrm{homo}}^{\mathrm{soft}}$ is essentially identical to the original linear phase ramp; when $r_{m} \gg R_{m}$, $w\left( r \right) \approx0$ and the $\frac{1}{{r_{m}}^{2}}$ far-field decay is recovered. Only within a narrow annulus of width $\Delta$ around $r \approx R_{m}$ is the original “broken-line” profile rounded, making both the phase and its radial derivative continuous and substantially suppressing high-frequency components at the edge.

For the vortex disk, the same soft-edge function was applied as Eq. (SM13):

$$\begin{aligned} \phi_{\text{vortex}}^{\text{soft}}\left( r_{m} \right)=w\left( r_{m} \right)C\left( R_{m}-r_{m} \right)\#\left( \mathrm{SM}13 \right) \end{aligned}$$

This localized smoothing of the phase at the disk edge makes the model more consistent with the finite domain-wall width and finite imaging resolution of real samples, while effectively suppressing Gibbs ringing in the FFT. As a result, subsequent evaluations of the forward model and its inversion algorithms can focus more directly on the underlying physical modeling, instead of being dominated by numerical artifacts arising from idealized geometric boundaries.

In the numerical simulations, the field of view was set to 128 nm, with the homogeneously magnetized disk and magnetic vortex disk radii fixed at 16 nm and 8 nm, respectively. To model the gradual decay of magnetization from the disk interior to the surrounding vacuum, $\Delta$ of 6 nm and 2 nm were applied to the homogeneously magnetized disk and magnetic vortex disk, respectively. The magnetic phase was computed using a standard electron phase-shift model. All holograms were stored as float 32-bit TIFF files.

**2. Supplementary results**

**2.1. Downsampling Strategy Comparison**

Fig. SM3 compares Fourier spectra of denoised holograms produced using different downsampling strategies: *anti-aliasing*, stride convolution, average pooling, and max pooling.

The noisy input hologram and its corresponding FFT show sideband peaks contaminated by shot noise. After denoising, the *anti-aliasing* approach preserves clean, well-defined sideband peaks with minimal spectral contamination. In contrast, stride convolution and average pooling produce noticeably weaker sidebands with residual noise, while max pooling introduces severe *aliasing* artifacts manifested as spurious spectral components scattered throughout the frequency domain. These results confirm that conventional downsampling operations violate the Nyquist–Shannon ^17^ sampling theorem, causing high-frequency folding that corrupts phase-encoded sideband information critical for holographic reconstruction.

**Fig. SM3. Comparison of Fast Fourier Transform (FFT) spectra for different downsampling strategies applied to a noisy hologram.**

**2.2. Denoising Performance of HoloDenoiser on Simulated *p–n* Junction Holograms**

Fig. SM4 presents spatial images and line profile analyses for simulated *p–n* junction holograms at dose = 1 e⁻/pixel. The noisy hologram and its reconstructed phase map exhibit severe shot noise contamination that obscures the underlying fringe pattern and phase gradient across the junction. After processing with HoloDenoiser, the denoised hologram shows clearly resolved interference fringes, and the corresponding phase map reveals a smooth, continuous phase transition characteristic of the built-in potential. Line profiles extracted along vertical (Profile A) and horizontal (Profile B) directions further demonstrate denoising fidelity. In the intensity profiles (Fig. SM4c), denoised results (blue circles) closely follow ground truth (red lines), successfully recovering the sinusoidal fringe modulation from heavily corrupted noisy data (gray dots). Phase profiles (Fig. SM4d) confirm that HoloDenoiser accurately reconstructs both the gradual phase ramp across the junction (Profile A) and the constant phase in uniform regions (Profile B), with denoised values exhibiting excellent agreement with ground truth. We conduct ablation studies to evaluate the contribution of each module within HoloDenoiser, and the results are summarized in Supplementary Table SMI.

**2.3. Validation on Experimental Vacuum Data**

Fig. SM5 shows the noise reduction factor (𝛿𝜑 before/after denoising) as a function of electron dose for both Linear and Counting modes at two dose rates. Despite the distinct noise characteristics between the two detector modes, HoloDenoiser consistently achieves a noise reduction factor of 3–4 fold across the full dose range of 1–200 e⁻/pixel, with slightly higher values (~4 fold) at the lowest doses where raw phase noise is most severe.

**2.4. Denoising Performance for Weak Magnetic Phases**

Fig. SM6 compares the two aperture radii used most often in practice, *q_c_* /3 and *q_c_* /2, applied to the same noisy and denoised holograms of Fig. SM6a. The Fourier spectrum in Fig. SM6b shows how each aperture selects the sideband, and the masked sidebands in the right panels make it clear that the denoised hologram keeps the same sideband peak as the noisy one while its surrounding background drops.

Fig. SM6c places the noisy and denoised reconstructions side by side for each aperture. Widening the aperture from *q_c_* /3 to *q_c_* /2 admits higher spatial frequencies, but in the noisy reconstruction it also lifts the background, since more high-frequency noise gets through. After denoising the background stays low at both radii, so the weak-phase feature stands out cleanly whatever the aperture size. The aperture-limited resolution belongs to the reconstruction, not to the network. Denoising only lowers the noise beneath that ceiling.

Fig. SM7 systematically evaluate denoising performance on simulated magnetic phase signals spanning a range of phase shift and electron doses. For homogeneously magnetized disks at a fixed dose of 1 e⁻/pixel, the weakest phase shift (~0.1 rad) produces raw noise fluctuations exceeding ±1.0 rad, yielding SNR < 1 where the magnetic structure is visually indistinguishable from background noise. After denoising, the phase profile accurately reproduces ground truth, with phase precision improving by approximately an order of magnitude. For higher phase shifts, denoised and ground truth profiles become virtually indistinguishable, demonstrating robust performance across the tested amplitude range.

**Fig. SM4.** **Denoising results for simulated *p–n* junction hologram (dose = 1 e⁻/pixel).** **a**, Noisy hologram (left) and denoised hologram (right) **b**, Corresponding phase map of **a**. **c**, Intensity distributions along line profile A and B, comparing noisy data (gray), ground truth (red solid line), and denoised results (blue open symbols). **d**, Phase distributions along line profile A and B.

**Fig. SM5.** **Noise reduction factor of HoloDenoiser as a function of electron dose for Linear mode (blue circles, upper panels) and Counting mode (purple triangles, lower panels).** The left column corresponds to a dose rate of 10 e⁻/pixel/s and the right column to 20 e⁻/pixel/s, both with exposure times ranging from 0.1 to 10 s. The noise reduction factor is defined as the ratio of the phase noise standard deviation before and after denoising. Across the full dose range examined (1–200 e⁻/pixel), the denoising performance remains robust across both detector modes.

TABLE SMI

Ablation of the HoloDenoiser on the Simulated Hologram Dataset (dose=1 e⁻/pixel)

| Mode | Description | PSNR | SSIM | 𝛿𝜑 |
| --- | --- | --- | --- | --- |
| HoloDenoiser | all components | 31.54±0.62 | 0.99±0.01 | 0.031±0.01 |
| w/o spectral prior | remove all spectral prior (Spectral channel; Mask; Loss) | 28.30±0.81 | 0.99±0.01 | 0.065±0.01 |
| replace FFC | replace FFC blocks with Conv | 27.64±1.08 | 0.98±0.01 | 0.061±0.03 |
| w/o WA+GDFN | without window attention and GDFN | 28.74±0.45 | 0.96±0.01 | 0.048±0.02 |
| w/o overlapping | use standard windows and without Gaussian reconstruction | 29.45±0.75 | 0.96±0.01 | 0.036±0.02 |
| replace fusion block | replace fusion block with element-wise addition | 28.82±1.53 | 0.99±0.01 | 0.042±0.02 |

**Fig. SM6. Sideband-aperture phase reconstruction for a magnetic vortex disk (dose = 20 e⁻/pixel). a**, Noisy and denoised off-axis holograms. Red boxes mark the enlarged fringe regions shown in the insets. **b**, Fourier spectrum of the noisy hologram with two sideband apertures overlaid: the blue and red circles correspond to radii of *q_c_* /3 and *q_c_* /2, respectively. The right panels show the corresponding masked sidebands for the noisy and denoised holograms. c, Reconstructed phase figure using the two apertures. The left half is reconstructed from the noisy hologram and the right half from the denoised hologram.

**Fig. SM7. Denoising and reconstruction of weak phase in homogeneously magnetized disk under different phase shift. a**, Reconstructed phase maps. The electron dose is fixed at 1 e⁻ /pixel, and the phase shift increases from ~0.1 to 1.56 rad from top to bottom. For each row, the leftmost column displays the raw noisy phase map, while the subsequent columns show the HoloDenoiser-denoised phase maps and ground truth maps, respectively. **b**, One-dimensional phase profiles extracted along the dashed line in **a**. The gray curves denote noisy reconstructions, red dashed curves show the ground truth, and the blue curves with circle markers correspond to the HoloDenoiser outputs.

**References**

1	Tamaoka T et al. Denoising electron holograms using the wavelet hidden markov model for phase retrieval—applications to the phase-shifting method. *AIP Advances* **11**, 025135 (2021).

2	Anada S, Nomura Y, Yamamoto K. Enhancing performance of electron holography with mathematical and machine learning–based denoising techniques. *Microscopy* **72**, 461-484 (2023).

3	Dabov K, Foi A, Katkovnik V, Egiazarian K. Image denoising by sparse 3-d transform-domain collaborative filtering. *IEEE Transactions on image processing* **16**, 2080-2095 (2007).

4	Zhang K, Zuo W, Chen Y, Meng D, Zhang L. Beyond a gaussian denoiser: Residual learning of deep cnn for image denoising. *IEEE Transactions on Image Processing* **26**, 3142-3155 (2017).

5	Lehtinen J et al. Noise2Noise: Learning image restoration without clean data. In Proceedings of the 35th International Conference on Machine Learning (2018).

6	Krull A, Buchholz T-O, Jug F. Noise2void-learning denoising from single noisy images. In Proceedings of the IEEE/CVF conference on computer vision and pattern recognition (2019).

7	Liang J et al. SwinIR: Image restoration using swin transformer. In 2021 IEEE/CVF International Conference on Computer Vision Workshops (ICCV) (2021).

8	Zamir SW et al. Restormer: Efficient transformer for high-resolution image restoration. In 2022 IEEE/CVF Conference on Computer Vision and Pattern Recognition (CVPR) (2022).

9	Ronneberger O, Fischer P, Brox T. U-net: Convolutional networks for biomedical image segmentation. In International Conference on Medical image computing and computer-assisted intervention (2015).

10	Zhang R. Making convolutional networks shift-invariant again. In Proceedings of the 36th International Conference on Machine Learning (2019).

11	Zou X, Xiao F, Yu Z, Li Y, Lee YJ. Delving deeper into anti-aliasing in convnets. *International Journal of Computer Vision* **131**, 67-81 (2023).

12	Chi L, Jiang B, Mu Y. Fast fourier convolution. In Advances in Neural Information Processing Systems (2020).

13	Chen Z, Badrinarayanan V, Lee C-Y, Rabinovich A. Gradnorm: Gradient normalization for adaptive loss balancing in deep multitask networks. In Proceedings of the 35th International Conference on Machine Learning (2018).

14	Kendall A, Gal Y, Cipolla R. Multi-task learning using uncertainty to weigh losses for scene geometry and semantics. In 2018 IEEE/CVF Conference on Computer Vision and Pattern Recognition (2018).

15	Cai Z et al. Exponential moving average normalization for self-supervised and semi-supervised learning. In 2021 IEEE/CVF Conference on Computer Vision and Pattern Recognition (CVPR) (2021).

16	Caron J. Model-based reconstruction of magnetisation distributions in nanostructures from electron optical phase images. PhD thesis, Dissertation, RWTH Aachen University (2017).

17	Shannon CE. Communication in the presence of noise. *Proceedings of the IRE* **37**, 10-21 (1949).
